# CP32, a novel anti‐oomycete cyclic peptide, interferes with cell‐wall deposition in *Phytophthora infestans*

**DOI:** 10.1111/jipb.70343

**Published:** 2026-07-16

**Authors:** Demetrio Marcianò, Stefano Rosa, Elena Marone Fassolo, Chiara Bertaso, Andrea Tagliani, Nina Gauri Capra, Federico Ballabio, Chiara Mizzotti, Giulia Rizzi, Sadia Fida Ullah, Lisa Kappel, Lucia Feni, Luca Tadini, Louise Jane Gourlay, Rosaria Russo, Vincent Bulone, Sabrina Pricl, Sandrine Ongeri, Sara Pellegrino, Carlo Camilloni, Silvia Laura Toffolatti, Vaibhav Srivastava, Paolo Pesaresi, Simona Masiero

**Affiliations:** ^1^ Dipartimento di Bioscienze, Università degli Studi di Milano Milano 20133 Italy; ^2^ Division of Glycoscience, Department of Chemistry, School of Engineering Sciences in Chemistry, Biotechnology and Health Royal Institute of Technology (KTH) Stockholm SE 106 91 Sweden; ^3^ Dipartimento di Scienze Farmaceutiche Università degli Studi di Milano Milano 20133 Italy; ^4^ Dipartimento di Fisiopatologia Medico‐Chirurgica e dei Trapianti Università degli Studi di Milano Milan 20100 Italy; ^5^ College of Medicine and Public Health Flinders University, Bedford Park Campus Adelaide 5042 South Australia Australia; ^6^ Molecular Biology and Nanotechnology Laboratory (MolBNL@UniTS), DEA University of Trieste Trieste 34127 Italy; ^7^ Department of General Biophysics, Faculty of Biology and Environmental Protection University of Lodz Łódź 90‐236 Poland; ^8^ Université Paris‐Saclay, CNRS, BioCIS Orsay 91400 France; ^9^ Dipartimento di Scienze Agrarie e Ambientali—Produzione, Territorio e Agroenergia Università degli Studi di Milano Milano 20133 Italy

**Keywords:** cellulose synthase targeting, cell wall integrity, cyclic peptide, *Phytophthora infestans*

## Abstract

*Phytophthora infestans*, the causal agent of late blight in potato and tomato, is a historically devastating pathogen that continues to pose a major threat to global food security. Although conventional fungicides remain a cornerstone of crop protection, growing concerns over their environmental impact and long‐term sustainability have driven the search for safer and more selective alternatives. In this context, next‐generation fungicides with high specificity and minimal ecological footprint are urgently needed. This study introduces CP32, a small cyclic peptide, identified via yeast two‐hybrid screening of a genetically encoded combinatorial peptide library, based on its specific interaction with the catalytic domain of the *P. infestans* enzyme Cellulose Synthase 2. CP32 exhibits anti‐oomycete activity, effectively inhibits sporangial growth at low micromolar concentrations, and significantly reduces late blight symptoms on tomato leaves and entire plants. *In silico* docking analyses, supported by molecular and biochemical evidence, confirm that CP32 compromises cell‐wall integrity by interfering with cellulose deposition and altering glucan composition. Furthermore, CP32 readily permeates the *Phytophthora* membrane and displays oomycete‐static properties, while showing no detectable toxicity toward non‐target organisms, such as bacteria, yeast, or plants. Collectively, these findings identify CP32 as a promising, targeted anti‐oomycete agent with a unique mode of action, targeting the *P. infestans* cell wall, representing a valuable contribution to the development of sustainable strategies for the control of late blight.

## Graphical abstract

The small cyclic peptide CP32 effectively inhibits *Phytophthora infestans*, the causal agent of potato and tomato late blight. Combined biochemical and phenotypic analyses demonstrate that CP32 disrupts cell wall biosynthesis, a key process in pathogen infection, resulting in a marked reduction of disease symptoms in plants.

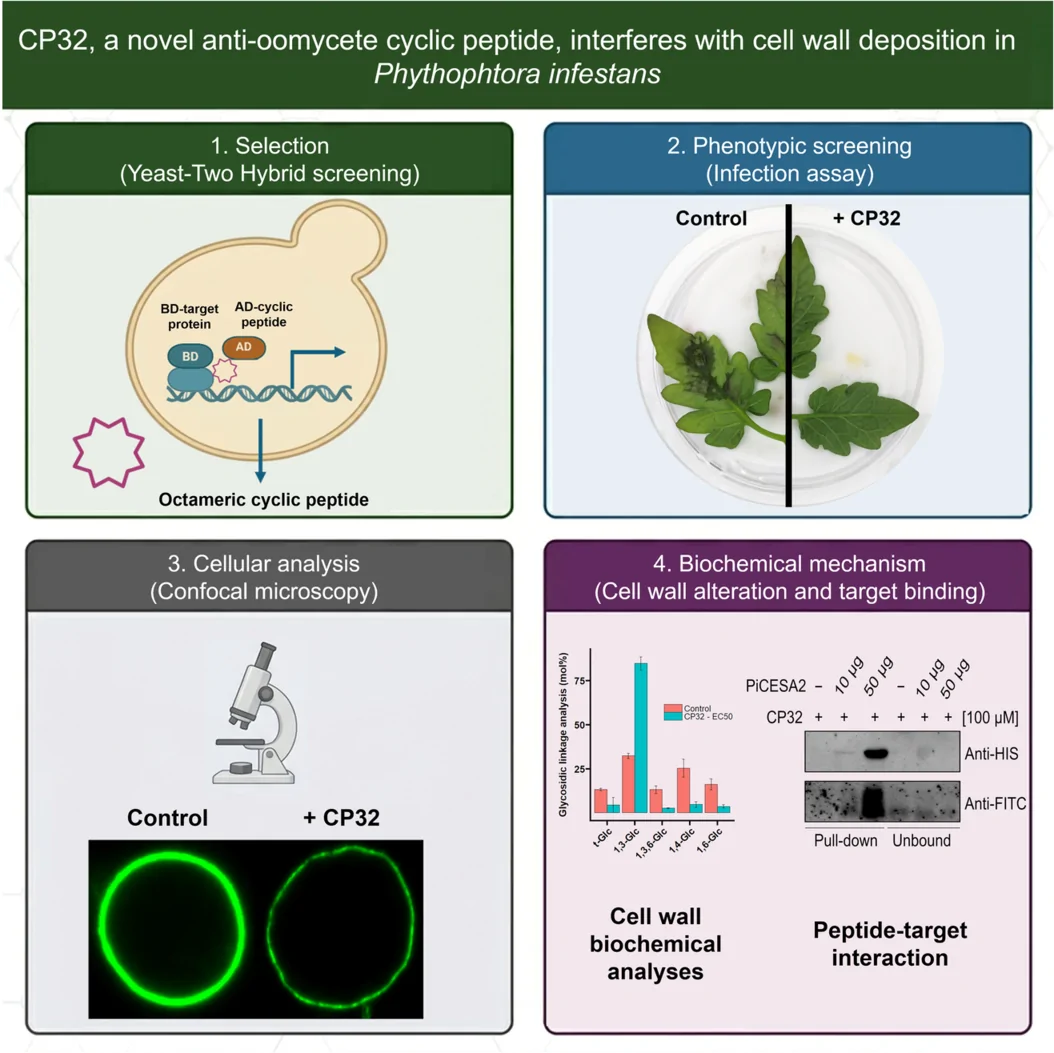

## INTRODUCTION

The oomycete phylum encompasses several genera widely recognized for causing devastating diseases in numerous plants and cultivated crops. Unless adequately controlled, oomycete‐borne diseases can result in substantial losses in yield, with concomitant effects on food availability and access to staple crops (Kamoun et al., 2015), thus posing a serious threat to global food security (Derevnina et al., 2016). The remarkable versatility of these organisms constitutes an ongoing challenge for disease management, as they swiftly develop resistance to fungicides and rapidly evade the actions of plant disease‐resistance genes (Derevnina et al., 2016). Among phytopathogenic oomycetes, *Phytophthora infestans* (Mont.) de Bary is a highly destructive and extensively studied oomycete species. It is responsible for late blight disease in both tomato (*Solanum lycopersicum*) and potato (*Solanum tuberosum*), leading to billions of dollars of annual economic losses (Judelson and Blanco, 2005; Haverkort et al., 2009).

Despite the widespread adoption of integrated plant‐disease management strategies—such as pathogen population monitoring, improved agronomic practices, and the use of genetic and biological resources—control of oomycete‐borne diseases is still largely dependent on fungicide application, which remains the most effective control method available (Volynchikova and Kim, 2022). However, the extensive use of chemicals for plant protection over the past 60 years has led to significant negative effects on both the environment and human health (Ahmad et al., 2024). These concerns have prompted several international agencies to call for a reduction in chemical pesticide use, and the concomitant development of sustainable alternative strategies that protect crops while minimizing the environmental and health impacts of conventional pesticides (Rennick et al., 2024). In recent years, peptide‐based agrochemicals have emerged as promising alternatives to conventional pesticides, owing to their ability to disrupt the activities of specific pathogen‐derived proteins, thus resulting in antibiotic compounds with a low environmental impact (Rosa et al., 2022, 2023; Zhang et al., 2023; Marone Fassolo et al., 2024). Genetically encoded libraries, in combination with the yeast two‐hybrid (Y2H) methodology, enable the rapid identification of short peptides that selectively bind to target proteins. This approach provides a powerful platform for the development of inhibitors that are specific for plant pathogens, with cell‐wall enzymes emerging as particularly promising targets (Grenville‐Briggs et al., 2008; Rzeszutek et al., 2019; Colombo et al., 2020; Ullah et al., 2026). The cell wall of *Phytophthora* species consists largely of polysaccharides, especially 1,4‐ and 1,3‐linked glucans. Among these, cellulose (1,4‐glucan) accounts for more than 40% of total cell‐wall composition and plays a crucial role in the life cycle of these organisms (Grenville‐Briggs et al., 2008; Mélida et al., 2013; Klinter et al., 2019).

Cellulose is synthesized by cellulose synthase (CESA) enzymes—transmembrane proteins that belong to glycosyltransferase family 2 (Lombard et al., 2014). These enzymes catalyze cellulose biosynthesis *via* a specialized intracellular catalytic domain and export the polymer through a transmembrane channel. In *P. infestans*, four different cellulose synthase genes (*PiCesA1*–*4*) have been identified, and inhibition of their biosynthesis, by gene silencing or chemical interference, significantly reduces the pathogenicity of *P. infestans* (Grenville‐Briggs et al., 2008; Gisi et al., 2019). Furthermore, functional analysis of the *Phytophthora capsici Pc*CESA1 enzyme has confirmed its essential role in mycelial growth, cystospore germination, and pathogenicity (Pang et al., 2020).

This study presents the discovery of CP32, a cyclic peptide that was identified by screening a genetically encoded yeast two‐hybrid library, using the *Phytophthora infestans* Cellulose Synthase 2 enzyme as bait. CP32 exhibits targeted anti‐oomycete activity against *P. infestans* and effectively suppresses late‐blight symptoms by weakening cell‐wall integrity. Phenotypic, biochemical, and molecular analyses corroborate the specific interaction between CP32 and its target protein, *Pi*CESA2, and thus support the potential of CP32 as a novel active ingredient for late‐blight management with an innovative mechanism of action.

## RESULTS

### CP32 mitigates the symptoms of late blight

To identify cyclic peptides (CPs) that interact with the catalytic domain of *Pi*CESA2 (amino acids 640 to 817), we leveraged CYCLIC, a combinatorial yeast two‐hybrid library, previously developed by Rosa et al. (2023), which contains approximately 5 × 10^6^ unique 8‐mer CPs.

From the initial screen, we selected 10 peptides that specifically interacted with the catalytic domain of *Pi*CESA2 and showed no detectable interaction with the remaining portion of the bait chimera, namely the GAL4 DNA‐binding domain (GAL4BD). All 10 hits were produced by microwave‐assisted solid‐phase peptide synthesis (Peqini et al., 2024; Table S1). To facilitate the cyclization process, a β‐alanine linker was incorporated into the sequence (Rosa et al., 2023). As β‐alanine is absent in the native peptide identified during screening, it was designated as position zero (0), preceding the eight amino‐acid residues present in the original sequence. Of the 10 hit peptides, CP32 emerged as the most effective, inhibiting the growth of *P. infestans* in liquid culture (Marcianò and Toffolatti, 2023) at micromolar concentrations (Table S2). Notably, EC_99_ (56.18 ± 8.54 μM) and EC_50_ (16.34 ± 2.35 μM) values determined in the growth inhibition assays (Figure 1A) were consistent with those obtained through the resazurin cell viability assay (EC_99_ = 46.69 ± 2.71 μM and EC_50_ = 19.21 ± 0.39 μM), highlighting effective anti‐oomycete activity below 50 μM (Figure 1A, B).

**Figure 1:**
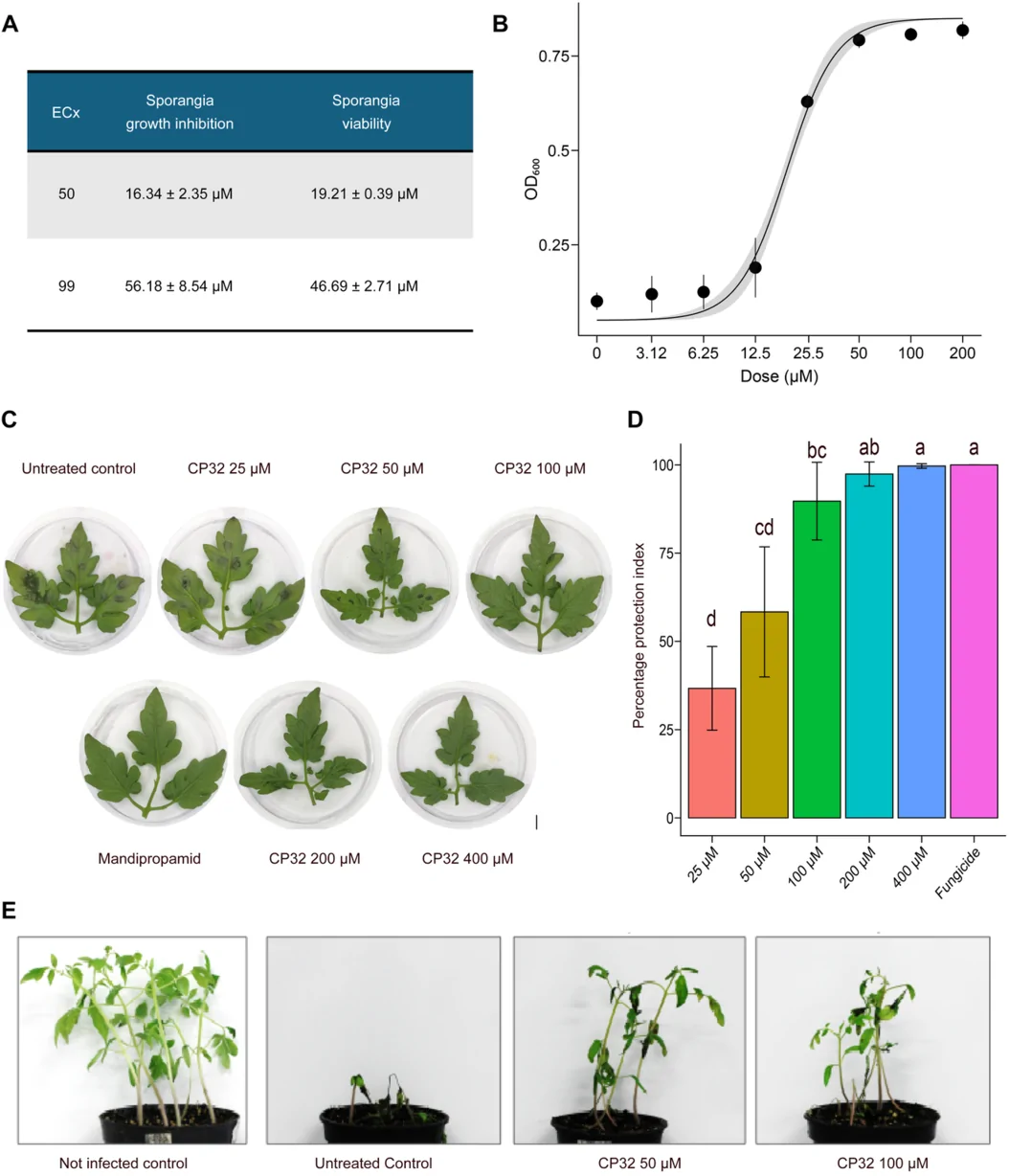
**CP32 inhibits the growth of** ***P*** ***hytophthora*** ***infestans*** **sporangia** **(A)** EC_50_ and EC_99_ values, calculated based on relative sporangial growth inhibition and sporangial viability in the presence of CP32, are expressed as μM ± *SE* (Standard Error). **(B)** Results of the sporangia cell‐viability assay using resazurin, a metabolic indicator of living cells. A shift from higher to lower absorbance values signals resazurin reduction and indicates the fraction of metabolically active fungal spores after 72 h. Points represent mean OD_600_ values obtained 3 d post inoculation (dpi); error bars represent standard deviations, and light gray ribbons depict the 95% confidence interval for the non‐linear regression model. **(C)** Images of tomato leaflets co‐inoculated *ex vivo* with *P. infestans* sporangia in either the absence (untreated control) or presence of different concentrations of CP32 (from 25 to 400 μM) and the fungicide mandipropamid (Scale bar 1 cm). **(D)** Quantitative evaluation of late blight symptoms (necrosis) on tomato‐leaf tissues at 7 d post‐inoculation (dpi). For each treatment, average values and standard deviations are reported. The different letters indicate significant differences between treatments, based on Kruskal–Wallis tests (*P* ≤ 0.05). **(E)** Images of tomato plants pre‐treated with CP32 (50 μM or 100 μM) and subsequently inoculated with *P. infestans* zoospore suspension. Images of CP32‐untreated and *P. infestans*‐untreated control plants are also shown.

To further explore CP32 anti‐oomycete properties, its ability to reduce tomato late blight symptoms in an *ex vivo* leaf bioassay was investigated. In untreated control samples, *P. infestans* successfully infected tomato leaflets, resulting in a disease severity index (DSI) of 61.88% ± 15.23%. In contrast, leaflets exposed to 10 mg/L of the commercial fungicide mandipropamid exhibited no late‐blight symptoms (Figure 1C, D). The comparison between DSI values was performed using the plant protection index (PPI; see Material and Methods), which quantifies the reduction in disease severity relative to the untreated control. The application of 100 μM CP32 to tomato leaves markedly reduced disease progression, exceeding 85% PPI (Figure 1C, D). Higher CP32 concentrations (200 and 400 μM) resulted in PPI values of 99.70% and 97.41%, respectively, demonstrating efficacy comparable to that of mandipropamid (10 mg/L) (Figure 1C, D). At lower concentrations, CP32 still exhibited anti‐oomycete activity—albeit reduced—with PPI values of 36.7% at 25 μM and 58.37% at 50 μM (Figure 1C, D). To further assess CP32 protection against late blight, we performed an *ex vivo* assay using potato tubers. Following inoculation with sporangia, no visible symptoms were observed in tubers treated with CP32 at concentrations above 100 μM (Figure S1). To assess the potential of CP32 as a novel commercial product, we also evaluated its ability to protect tomato plants grown under controlled conditions. Two concentrations (50 and 100 μM) were applied to tomato plants, and disease severity was assessed 7 d after infection (Table S2). All plants in the untreated control died following *P. infestans* infection. CP32‐treated plants at 100 μM exhibited a protection index of 68.9% ± 24.5%, while at 50 μM the protection index was 57.2% ± 18.3% (Figure 1E; Table S2).

In addition, CP32 was tested at 200 μM against three oomycete species related to *P. infestans* (Table S3). Specifically, *in vitro* growth inhibition exceeded 85% for *Pythium ultimum* (85.42% ± 7.28%) and for *Phytophthora capsici* (89.25% ± 7.35%) and provided strong protection on grapevine leaves inoculated with the biotrophic pathogen *Plasmopara viticola* (94.92% ± 6.45%), showing no significant difference when compared to mandipropamid effectiveness. All studied oomycetes possess CESA2 enzymes with conserved catalytic domains that are highly similar to the catalytic domain of *Pi*CESA2 (Figure S2).

Overall, these results show evident anti‐oomycete activities of CP32 under both *in vitro* and *in planta* conditions.

### CP32 perturbs *P. infestans* cell‐wall deposition and alters its composition

Since CP32 alleviates late blight symptoms at micromolar concentrations, we investigated whether its anti‐oomycete activity targets cellulose biosynthesis. We first assessed whether CP32 inhibits *de novo* cell wall deposition in regenerating *P. infestans* protoplasts. Protoplasts were treated with 25 μM CP32, a concentration selected based on dose–response curves to approximate the peptide's half‐maximal effective concentration (EC_50_), while avoiding toxic effects observed at higher doses. After 6 h incubation, protoplasts were incubated with GFP‐CBM3A, a chimeric protein, which specifically binds crystalline cellulose. CP32‐treated protoplasts showed notably reduced fluorescence intensity compared to untreated controls (Figure 2A, B), and quantification along the protoplast periphery confirmed a significant decrease in staining intensity (Figure 2C). Additionally, cell‐wall thickness was significantly reduced in CP32‐treated samples (Figure 2D).

**Figure 2:**
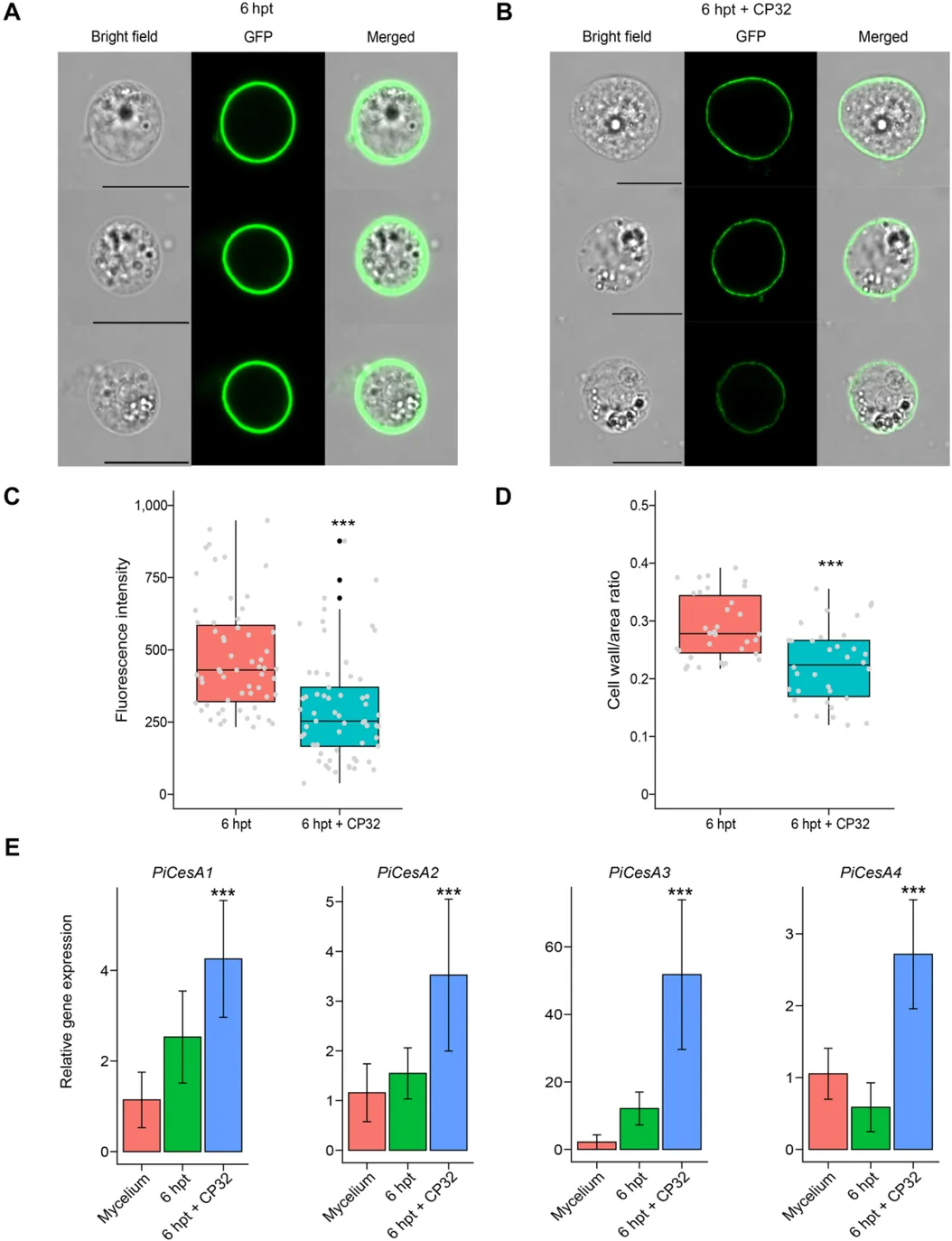
**Effects of CP32 treatment on the** ***de novo*** **cell‐wall deposition of** ***P*** ***hytophthora*** ***infestans*** **protoplasts** **(A**, **B)** Fluorescent images of three regenerated protoplasts after 6 h of enzymatic cell‐wall removal (6 h post‐inoculation, 6 hpt), stained with GFP‐CBM3A. **(A)** Untreated control (*n* = 62); **(B)** CP32‐treated (25 μM) samples (*n* = 62). Scale bar = 15 μm. **(C)** Quantification of fluorescence intensity at the cell periphery (*n* = 62 for each treatment) (*F* = 21.87; df = 1; *P*‐value < 0.001). **(D)** Ratio of the cell‐wall area to the total cell area, which serves as an indicator of cell‐wall density (*n* = 34 for each treatment) (*F* = 19.55; df = 1; *P*‐value < 0.001). Light gray values depict single observations. **(E)** Expression of *P. infestans CesA* genes in regenerating protoplasts after 6 h of enzymatic cell‐wall removal, either in the absence (6 hpt) or presence of 25 μM CP32 (6 hpt + CP32). The expression level of each *PiCesA* gene in the mycelial cDNA sample was set to 1.0. Values represent the means of three biological replicates, with error bars indicating the standard deviation. Statistical comparisons of relative expression values among different treatments were performed using one‐way ANOVA followed by Tukey's HSD test. Significant differences compared to the untreated control are marked by asterisks (*P < 0.05; **P < 0.01; ***P < 0.001).

Similar reductions in GFP‐CBM3A staining were observed in germinating sporangia (16 h post‐treatment) and encysted zoospores (0.5 h post‐treatment) treated with CP32 (Figure S3), indicating inhibitory effects on cell‐wall deposition in key infection structures across multiple developmental stages.

Consistent with these observations, RT‐qPCR analysis of regenerating protoplasts revealed significant upregulation of all four *PiCesA* genes 6 h after CP32 treatment (Figure 2E), with *PiCesA3* showing the strongest transcriptional response and likely representing the most abundant CESA enzyme, based on basal expression levels. This robust transcriptional compensation suggests that cells attempt to offset CP32‐mediated inhibition of cellulose production, consistent with a previously described compensatory mechanism (Fugelstad et al., 2009).

*In vivo* localization studies provided additional support for CP32–PiCESA2 interaction. CP32‐FITC was used to track CP32 localization in *P. infestans* germ tubes arising from sporangia, revealing that CP32‐FITC predominantly accumulates at the plasma membrane, as confirmed by colocalization with FM4‐64, a plasma membrane marker (Figure S4). Notably, the fluorescent signal is concentrated in discrete punctate structures at the membrane. These foci are hypothesized to correspond to protein complexes containing PiCESAs, providing indirect evidence for localized functional interactions between CP32 and the cellulose synthase machinery (Figure S4).

To determine whether these molecular findings translated into alterations in cell‐wall composition, *P. infestans* mycelium was exposed to 25 μM CP32 for 3 d. Absolute quantification of monosaccharides revealed a ~30% reduction in total glucose content (CP32‐treated samples: 2.67 μmol vs. control: 3.65 μmol) (Figure 3A). Glycosidic linkage analysis of alcohol‐insoluble fractions showed a striking ~80% reduction in 1,4–linkages associated with cellulose, accompanied by an increase in 1,3‐glucans, indicating a compensatory mechanism (Figure 3B). The 1,3‐glucans in CP32‐treated samples were predominantly linear with significantly reduced cross‐linking (lower 1,6‐ and 1,3,6‐linkages), suggesting fundamentally impaired cell‐wall architecture and structural integrity.

**Figure 3:**
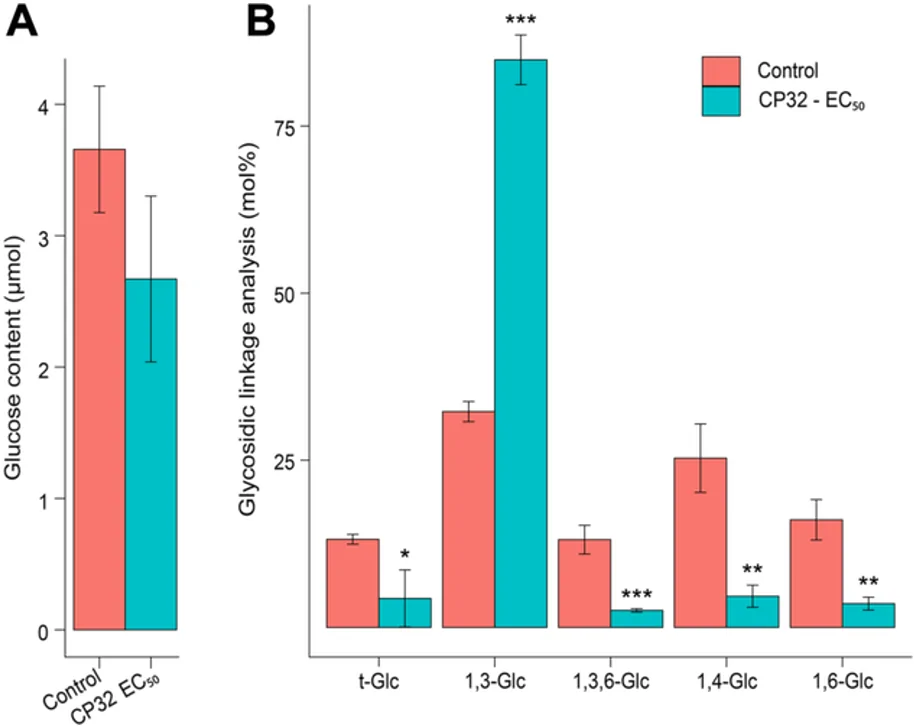
**Monosaccharide and polysaccharide composition of** ***P*** ***hytophthora*** ***infestans*** **mycelial cell walls after prolonged exposure to 25 μM CP32** **(A)** Absolute quantification (μmol) of glucose in the cell walls of *P. infestans* protoplasts that had been treated with 25 μM CP32 (light blue) and untreated controls (red) after 3 d of peptide exposure. **(B)** Glycosidic linkage analysis of purified mycelial cell walls (alcohol insoluble fraction, AIF) from CP32‐treated (light blue) and untreated (red) *P. infestans* samples. Values represent the means of three replicates, with error bars indicating the standard deviation. Significant differences compared to the untreated control, using the ANOVA test, are marked with asterisks (*P < 0.05; **P < 0.01; ***P < 0.001).

Despite the activation of compensatory mechanisms, suggested by upregulated *CESA* gene expression and increased synthesis of alternative glucans, these adaptations proved insufficient to overcome the severe alterations to cell‐wall structure induced by CP32 inhibition of *Pi*CESA2. The failure of these protective responses explains the potent anti‐oomycete efficacy observed at both the cellular and organism levels, demonstrating that CP32‐mediated disruption of *Pi*CESA2 function represents a promising antimicrobial strategy.

### CP32 interacts with *Pi*CESA2 and inhibits its enzymatic activity

To demonstrate the specificity of CP32 toward *Pi*CESA2, we performed Y2H assays using the catalytic domains of all four *P. infestans* CESA enzymes. CP32 showed a specific interaction with *Pi*CESA2, while no interaction was detected with *Pi*CESA1, *Pi*CESA3, or *Pi*CESA4 (Figure S5). Although a slight autoactivation was observed for *Pi*CESA4, which somewhat limits the conclusiveness of that result, the overall data clearly support the selective interaction of CP32 with *Pi*CESA2. To assess the direct inhibition of *Pi*CESA2 catalytic activity, we expressed full‐length *Pi*CESA2 in *Saccharomyces cerevisiae* using a well‐established system (Drew et al., 2008), incorporating an N‐terminal His_8_‐yEGFP tag. Enzymatic activity was quantified by measuring UDP release from a UDP‐glucose substrate (basal activity: 99.5 μM UDP mg^−1^ protein h^−1^), as previously described (Drew et al., 2008; Pang et al., 2020). While complete inhibition was not achieved at the tested concentrations, CP32 caused a significant reduction in enzymatic activity (Figure S6A, B).

CP32 binding to *Pi*CESA2 was further validated by NiNTA pull‐down assays using FITC‐labeled CP32 and purified 6×His::*Pi*CESA2 fragment (residues 318–770). CP32 was recovered in a dose‐dependent manner with increasing amounts of *Pi*CESA2 (0, 10, and 50 μg), while no signal was detected in the absence of protein, confirming a specific interaction (Figure S6C).

To quantify CP32‐*Pi*CESA2 binding affinity, we performed microscale thermophoresis (MST) with fluorescently labeled *Pi*CESA2, at increasing CP32 concentrations (0.8 μM–12.5 mM). Although binding was detected, the apparent dissociation constant was in the millimolar range (*K*_*d*_ = 7.89 ± 4.09 mM), and binding saturation could not be achieved due to CP32 solubility limitations (Figure S6D). This low apparent binding affinity likely reflects the use of a truncated *Pi*CESA2 domain that lacks the native, membrane‐embedded architecture required for high‐affinity interactions.

### Docking studies indicate that CP32 binds the *Pi*CESA2 TED motif

The model of the *Pi*CESA2 GT (glycosyltransferase) domain, predicted by AlphaFold2 (Figure 4A, B), exhibits strong structural similarities to the cellulose/chitin synthase catalytic domains of other species, including *Phytophthora sojae* (PDB 7WJO), *Gossypium hirsutum* (PDB 7D5K), *Populus tremula* (PDB 8G2J), and *Cereibacter sphaeroides* (PDB 5EJ1). Although sequence identity levels between the *P. infestans* GT domain and its homologs are relatively low (13.5% for *P. sojae*, 22% for *G. hirsutum*, 20.8% for *P. tremula*, and 35.4% for *C. sphaeroides*), structural alignments performed with Foldseek yielded a probability score of 1.0, indicating 100% confidence in the predicted model's structural accuracy. This suggests that, despite low sequence identity, the structural conformation of the GT domain is highly conserved across these species.

**Figure 4:**
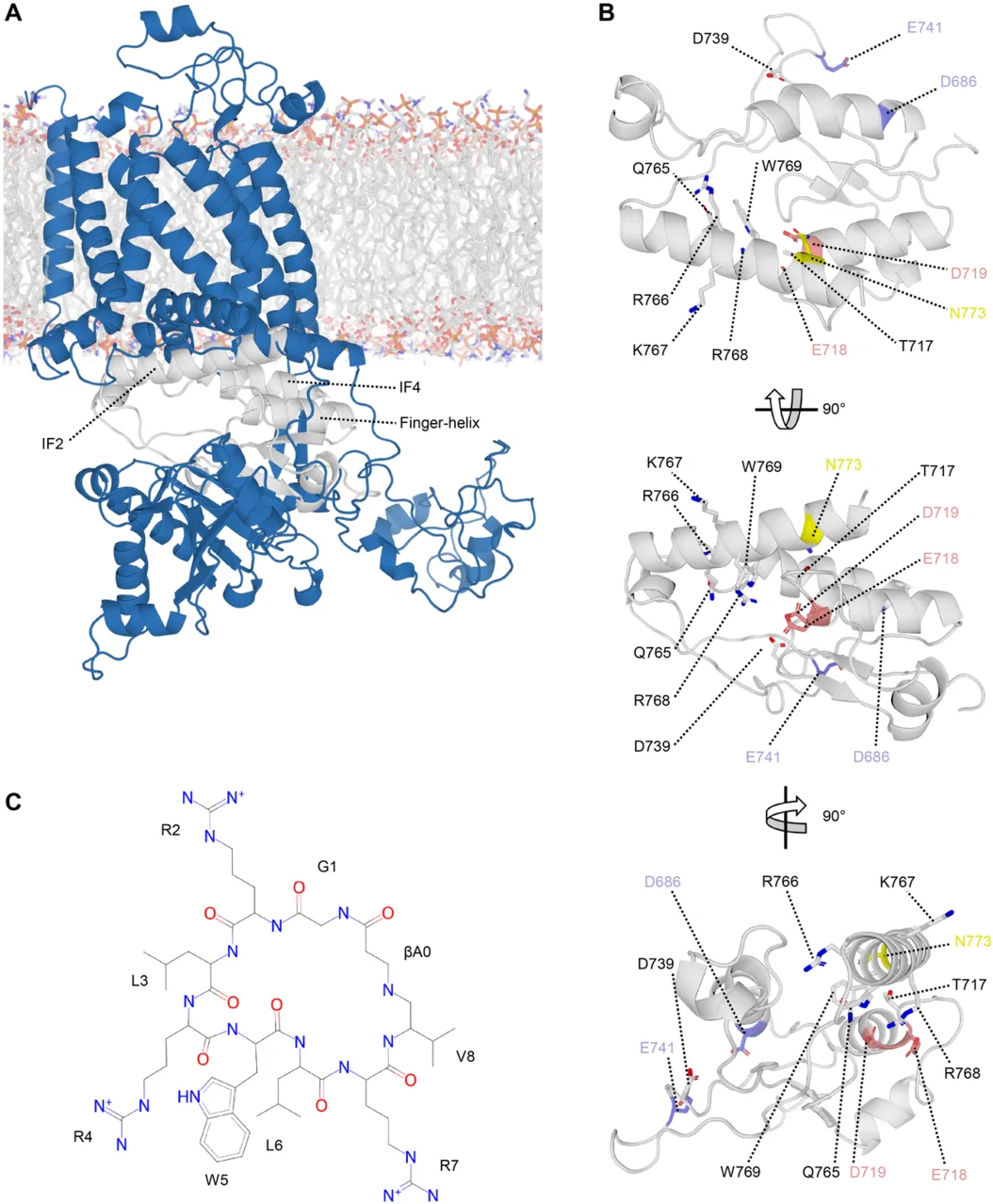
***In silico*** **models of** ***Pi*** **CESA2 and the cyclic peptide CP32** **(A)** Predicted model of *Pi*CESA2 (residue range 197–1,026). The GT domain is colored in light gray, the rest of the protein in blue. The protein is inserted into a POPE‐POPC membrane represented by sticks, with carbon (C) atoms shown in light gray, phosphorus (P) atoms in orange, oxygen (O) atoms in red, and nitrogen (N) atoms in blue. **(B)** Predicted model of the GT domain of *Pi*CESA2 (residues 644–778), colored in light gray. Residues Q765, R766, K767, R768, and W769 of the QRKRW B2‐motif in the IF2 helix, residue T717 of the TED motif in the finger helix, and residue D739 of the B1 motif in the flexible region are shown as sticks, with C atoms in light gray, O atoms in red, and N atoms in blue. Residues E718 and D719 of the TED motif, mutated in mutant M1 to Q718 and N719, are shown as sticks, with C atoms in pink, and O atoms in red. Residue N773 of the IF2 helix, mutated in mutant M2 to A773, is shown as sticks, with C atoms in light yellow, O atoms in red, and N atoms in blue. Residues D686 and E741 of the IF4 helix and the flexible region, respectively, which were mutated in mutant M3 to N686 and Q741, are shown as sticks with C atoms in purple, and O atoms in red. **(C)** Two‐dimensional conformation of the skeletal formula of the cyclic peptide CP32, cyclo (βAla‐Gly1‐Arg2‐Leu3‐Arg4‐Trp5‐Leu6‐Arg7‐Val8). In all stick representations, backbone and hydrogen (H) atoms are omitted.

In the light of these findings, a comparative structural analysis with *C. sphaeroides* cellulose synthase, exhibiting the highest sequence identity and an E‐value of 2.40e−8, indicated a statistically significant match between the query and target structures. Notably, this selected structure corresponds to the bacterial cellulose synthase BcsA–BcsB complex, which contains a cellulose polymer. This provides valuable insight not only into the GT domain's position within the overall structure, but also into its interaction with the elongating polysaccharide (Figure S7A).

An iterative, sequence‐independent, structural alignment generated by PyMOL revealed that backbone atoms (N, Ca, C, O) of the *Pi*CESA2 GT domain model (residues 647–778) exhibit a root‐mean‐square deviation (RMSD) of 1.13 Å compared to the backbone atoms of *C. sphaeroides* BcsA (residues 266–392). Interestingly, both structures share key residues at the entrance to the transmembrane (TM) channel. This region is defined by the amphipathic helix known as “IF2” (Morgan et al., 2013, 2014, 2016), which harbors the B2 motif “QXXRW” (Grenville‐Briggs et al., 2008) that forms the acceptor‐binding site (Figures 4B, S7B). In addition, the channel gate is defined by the “finger‐helix”, which contains the conserved threonine‐glutamate‐aspartate (TED) motif. These residues interact with the acceptor end of the translocating cellulose polymer and UDP‐glucose glycosyl donors (Morgan et al., 2013, 2014, 2016; Figures 5B, S7). To identify potential CP32 binding sites, a blind docking analysis was conducted between the *Pi*CESA2 GT domain and CP32. This approach identified three candidate binding regions within the GT domain (Figure S8): (i) the channel entrance; (ii) the space enclosed by the IF2 helix and a second helix spanning residues A675−Y688 (termed IF4 for convenience); and (iii) the flexible region containing the conserved residue D739, which is part of the B1 motif (Grenville‐Briggs et al., 2008). The top‐scoring docking poses for these regions yielded Schrödinger Maestro GlideXP scores of −11.964, −10.322, and −10.600, respectively. Given the relative proximity between these values, none of the potential sites could be excluded a priori. To validate and map the CP32 binding site(s), we therefore designed three sets of *Pi*CESA2 mutations, each featuring specific amino‐acid substitutions to disrupt or compromise the CP32 interaction at a particular site (Figure 5A). The targeted residues and corresponding conservative mutations were E718Q and D719N (TED motif, M1) for the channel entrance, N773A for the IF2–IF4 region (M2), and D686N and E741Q for the flexible region (M3). To test the interaction between the cyclic peptide CP32 and the three *Pi*CESA2 mutant variants, a Y2H assay was performed as reported in Figure 5B. It revealed that CP32 interacts with the wild‐type *Pi*CESA2 and with the M2 (N773A) and M3 (D686N/E741Q) *Pi*CESA2 variants, but not with the M1 (E718Q/D719N) variant of the catalytic TED domain (Figure 5A, B).

**Figure 5:**
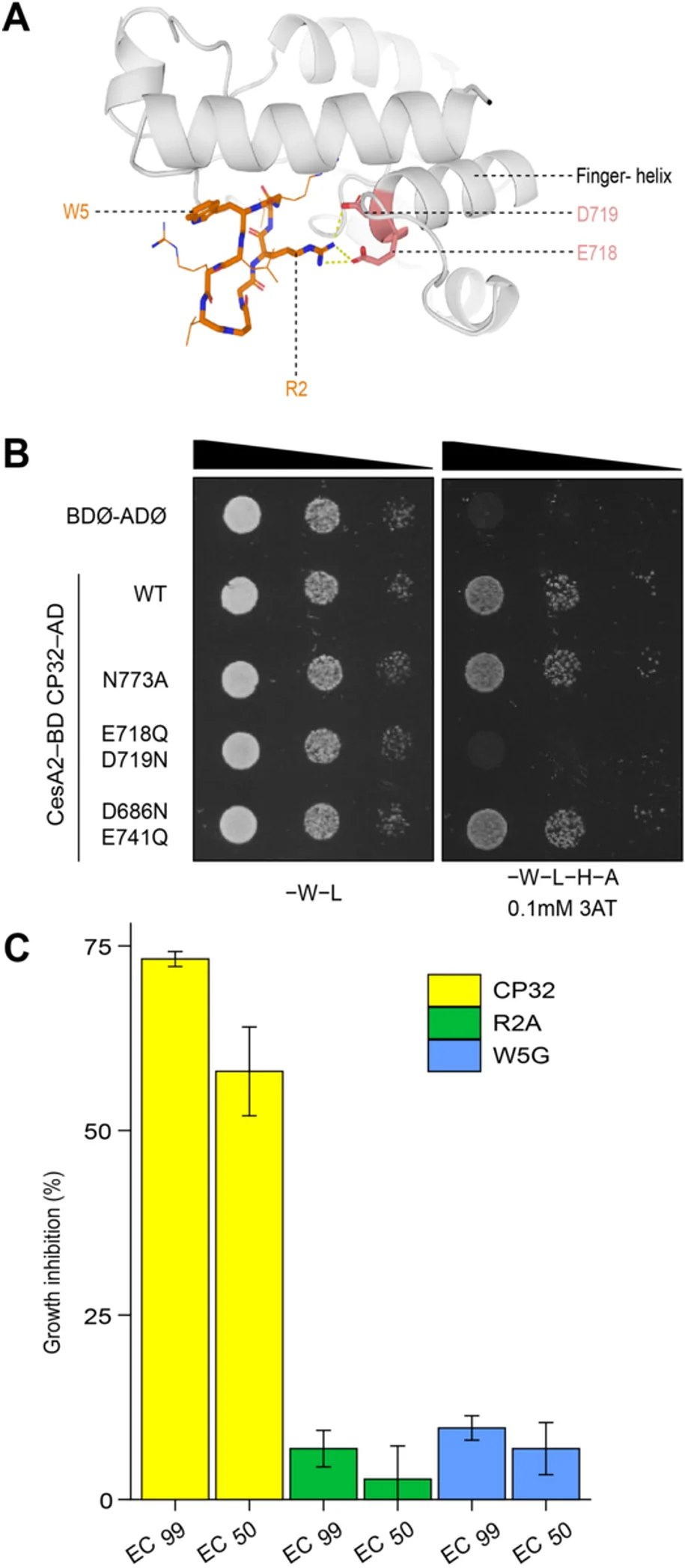
**Validation of the predicted CP32 docking site on the** ***Pi*** **CESA2 GT domain** **(A)** The most likely docking pose for the CP32 cyclic peptide is located at the channel entrance of the *Pi*CESA2 GT domain. The peptide backbone is depicted as sticks, with C atoms in orange, O atoms in red, and N atoms in blue. The side‐chains of the peptide are represented as lines, except for the side‐chains of residues R2 and W5, which are represented as sticks, with C atoms in orange, and N atoms in blue. The *Pi*CESA2 GT domain is represented in light gray cartoon with the TED motif highlighted in pink. The side‐chains of residues E718 and D719 are shown as sticks with C atoms in pink and O atoms in red. The H‐bonds established between the side‐chain of residue R2 of the cyclic peptide and the side‐chain of residues E718 and D719 of the *Pi*CESA2 GT domain are represented by yellow dashed lines. H atoms are not shown. **(B)** The *Pi*CESA2 mutant variants were generated and used in yeast two‐hybrid assays to verify their ability to physically interact with CP32. CP32 is still able to interact with N773A and D686N/E741Q, while the interaction is lost with the PiCESA2 variant E718Q and D719N. **(C)** CP32 mutant versions (R2A or W5G) lose their biological activity and are unable to arrest *Phytophthora infestans* sporangial growth. Their antimicrobial activity was calculated using resazurin viability assays and expressed as a percentage of growth inhibition.

The best docking pose of the CP32 cyclic peptide was identified at the channel entrance of *Pi*CESA2, and docking analyses indicated that the R2 and W5 residues of CP32 may play a pivotal role. To confirm these data, we mutated each of the CP32 residues to alanine and tested whether these variants retain their ability to interact with *Pi*CESA2 (Figure S8).

In addition, two CP32 variants carrying substitutions at positions R2 and W5 (CP32 R2A: βA‐GALRWLRV and CP32 W5G: βA‐GRLRGLRV) were chemically synthesized and tested for their anti‐oomycete activity, using the resazurin‐based assay, as previously described.

While alanine was systematically used in scanning mutagenesis to assess the contribution of each residue, specific substitutions were selected for anti‐oomycete activity assays based on docking analyses. R2 was mutated to alanine to reduce polar and electrostatic interactions due to the loss of the large positively charged arginine side chain. Conversely, W5 was mutated to glycine rather than alanine to better disrupt the hydrophobic interactions at this position, as glycine lacks a side chain and thus minimizes hydrophobic contacts. As expected, both peptide variants lost their anti‐oomycete activity (Figure 5C).

### CP32 crosses the *P. infestans* cell membrane and exhibits cytostatic effects

Antimicrobial peptides are generally cationic or amphipathic molecules that contain multiple arginine residues, which allow them to interact with the negatively charged phospholipids in the cell membranes of pathogens (Liu et al., 2025). To further characterize the mode of action of CP32, we assessed the ability of SYTOX Green—a DNA‐intercalating agent that cannot freely diffuse across the plasma membrane—to penetrate *P. infestans* hyphae in the presence of CP32. Notably, CP32 facilitates SYTOX Green uptake, likely by increasing the permeability of the *P. infestans* cell membrane, as shown by the intense green fluorescence observed in hyphae treated with CP32 (Figure S10). However, CP32 interaction with the plasma membrane does not lead to sporangial death, and sporangia exposed to CP32 for 3 d at EC_50_ concentration remain viable and develop mycelia upon peptide removal (Figure S11). Collectively, these results show that, despite the ability of SYTOX Green to permeate the plasma membrane in the presence of CP32, growth restoration following prolonged exposure excludes membrane disruption as part of its mechanism of action.

To further estimate the capacity of CP32 to permeate biological membranes, the *in vitro* PAMPA‐BBB assay was carried out. The measured effective permeability coefficient of CP32 was *P*_*e*,exp_ = (4.6 ± 0.3) × 10^−6^ cm/s. To contextualize this result, CP32 was assessed alongside three additional cyclic peptides, namely cyclosporin A (CSA), cyclosporin D (CSD), and enniatin B (EnB), under identical experimental conditions.

CP32 exhibited significantly higher permeability than either CSA [(2.1 ± 0.2) × 10^−6^ cm/s] or CSD [(0.93 ± 0.5) × 10^−6^ cm/s] and was only moderately less permeable than EnB [(8.3 ± 0.7) × 10^−6^ cm/s]. Based on established PAMPA‐BBB classification criteria (Di et al., 2003; Rosa et al., 2023; Table S4), CP32 can be categorized as an effective membrane‐permeable peptide, which accounts for its ability to reach its intracellular target.

### CP32 is highly specific and harmless to non‐target organisms

The specificity of CP32 was further evaluated to determine whether it might have potentially adverse effects on non‐target organisms. At its EC_99_ concentration, CP32 did not inhibit bacterial or yeast growth when added to the culture medium and did not show any cytotoxic effects on HT‐29‐immortalized human intestinal cells (Figure S12).

Moreover, to confirm the oomycete specificity of CP32 and rule out potential interference with cellulose accumulation in plant cells, we performed Y2H assays to assess whether CP32 interacts with cellulose synthase enzymes from tomato (*Sl*) and potato (*St*). Using the catalytic domains of *Sl*CESA‐like E1, *St*CESA2, and *Sl*CESA4, the assays showed no activation of the yeast HIS3 reporter gene, indicating that CP32 does not physically interact with these domains (Figure S5).

Furthermore, to rule out any adverse effect on plant growth, we applied CP32 to tomato leaves (Figure S13A). Seven d after treatment, no signs of stress were observed in treated leaves. Moreover, their photosynthetic activity, measured by PAM fluorometry, remained comparable to that of control leaves. In contrast, leaves treated with the herbicide glyphosate exhibited reduced photosynthetic activity.

We further evaluated the effects of CP32 following repeated applications (weekly for 3 consecutive weeks) on young tomato plants (2‐week‐old). CP32‐treated plants were compared with untreated controls receiving water alone (*n* = 15 and *n* = 12, respectively). At a concentration of 100 μM, CP32 did not cause any visible growth differences, nor did it induce necrosis or premature yellowing (Figure S14), confirming that CP32 does not affect tomato plant growth under these conditions. To determine whether CP32 affects CESAs required for primary cell‐wall formation, we assessed its impact on expanding plant cells. Seedlings of *Arabidopsis thaliana* were co‐cultivated with CP32, and hypocotyl growth—which relies exclusively on cell expansion rather than cell division—was measured. As a positive control, we used isoxaben (Ogden et al., 2024), a known inhibitor of cell expansion and cellulose synthases. As shown in Figure S13B, C, CP32 did not affect hypocotyl growth, confirming that it does not inhibit plant CESA enzymes required for primary cell‐wall formation.

## DISCUSSION

In recent years, there has been a marked increase in the use of peptides as tools for controlling plant diseases (Huang et al., 2021), including infections caused by oomycetes (Colombo et al., 2020). The use of short peptides as antimicrobials has been inspired by natural defense systems (Rosa et al., 2022), while genetically encoded combinatorial libraries have expanded our ability to identify peptides that interfere with the biological activity of selected protein targets. In this context, peptides that inhibit the biological functions of crucial enzymes, such as those involved in cell‐wall biosynthesis, represent an innovative approach to oomycete control (Colombo et al., 2020). This strategy aligns with the broader concept of protein interference, which encompasses the disruption or modulation of protein function *via* interactions with small peptides or protein fragments (Bruzzoni‐Giovanelli et al., 2018; Dickwella Widanage et al., 2024; Selim and Alva, 2024).

Since most *P. infestans* infections are driven by asexual spores (sporangia and zoospores) that require correct cell‐wall organization and cellulose biosynthesis for host penetration (Lacalendola et al., 2022), *PiCesA* gene products are ideal targets for the design of peptide inhibitors (Grenville‐Briggs et al., 2008; Li et al., 2022). In the present study, we identified CP32, a cyclic peptide that interferes with the activity of the *Pi*CESA2 enzyme. The application of CP32 limits late blight progression in both potato and tomato; furthermore, it retains antimicrobial activity on closely related species, such as *P. capsici*, *P. ultimum*, and *P. viticola*. This broad‐spectrum anti‐oomycete activity is enabled by the high conservation of CESA2 catalytic domains across these species, supporting the hypothesis that CP32 targets a conserved functional component of the oomycete cellulose biosynthesis machinery. Consistently, a similar broad‐spectrum efficacy has been observed for commercial Carboxylic Acid Amide (CAA) fungicides (Blum et al., 2026).

Additional Y2H analyses further corroborate the absence of physical interactions between CP32 and plant CESA homologs, consistent with the known evolutionary divergence between cellulose synthases in oomycetes and plants (Blum et al., 2010b; Colombo et al., 2020) and our toxicity assay results (Figures S12–S14).

Moreover, the physical interaction between *Pi*CESA2 and CP32 was demonstrated by a pull‐down assay and MST. Although it was not possible to accurately calculate the binding affinity due to technical limitations, binding was estimated to be in the millimolar range. With regards to catalytic activity, CP32 was shown to reduce *Pi*CESA2 activity by ~30% at 200 μM CP32. The differences observed between our *in vivo* and *in vitro* results that employ full‐length and truncated domain constructs, respectively, are likely due to incorrect protein folding and/or instability of the latter, and/or the requirement for *Pi*CESA2 to be in a more native context. This common limitation is well‐documented for large membrane‐associated glycosyltransferases, as previously reported for *P. capsici Pc*CESA1 (Pang et al., 2020).

The mode of action of CP32 was further supported by docking analyses, which show that it binds to the *Pi*CESA2 TED motif, which is highly conserved among processive glycosyltransferases. Specifically, the docking results indicated that arginine at position 2 of CP32 forms multiple hydrogen bonds with glutamate and aspartate residues within the TED motif. Consistent with these observations, point mutations of these residues (E718Q/D719N) disrupted CP32 binding. Reciprocally, mutations in CP32 at arginine‐2 (R2A) and tryptophan‐5 (W5G) abolished its interaction with wild‐type *Pi*CESA2 in Y2H assays (Figure S9), confirming the critical role of these residues for binding. In agreement with these findings, both CP32 variants exhibited significantly reduced anti‐oomycete activities in bioactivity assays (Figure 5C).

At a physiological level, the specific interference of CP32 with *Pi*CESA2 activity resulted in a global modification of the *P. infestans* cell wall composition. When applied at a partially effective concentration (25 μM), CP32 caused an approximately 80% reduction in cellulose accumulation, as shown by glycosidic linkage analysis (Figure 3B). This effect was accompanied by a significant increase in 1,3‐glucans and a simultaneous reduction in cross‐linking structures, specifically 1,6‐ and 1,3,6‐linked glucose, indicating that CP32 disrupts the overall glucan composition of the cell wall. The observed increase in other glucans likely reflects a compensatory response aimed at preserving the strength and structural integrity of the cell wall, as previously observed in related oomycete species (Fugelstad et al., 2009; Guerriero et al., 2010; Li et al., 2022). Indeed, rearrangement of the macromolecular architecture of cell walls is a well‐established phenomenon in both oomycetes and plants, which occurs in response to cell‐wall inhibitors as well as through point mutations in biosynthetic enzymes (Harris et al., 2012; Dickwella Widanage et al., 2024).

This rearrangement may be driven by the presence of a partially redundant set of four *CesA* genes in the *P. infestans* genome, which could confer resilience to the cellulose biosynthesis machinery. Among these, *PiCesA3* represents the most abundant *CesA* transcript in *P. infestans* and related species (Grenville‐Briggs et al., 2008; Pang et al., 2020), highlighting its potential key role in cellulose biosynthesis. However, despite its lower transcription level, the importance of *Pc*CESA1 for growth and pathogenicity has also been demonstrated (Li et al., 2022). Moreover, a previous study in the related species *P. capsici* has also demonstrated that *Pc*CESA1, *Pc*CESA2, and *Pc*CESA4 may associate to form a complex, potentially analogous to the cellulose synthase complexes observed in plants (Pang et al., 2020).

In agreement with this model, treatment with CP32 enhanced the transcription of all *CesA* genes during *de novo* cell‐wall deposition. However, despite this transcriptional upregulation, cell‐wall organization and composition remained severely impaired. Indeed, upregulation of the remaining *CesA*‐encoding genes (*PiCesA1*, *PiCesA3*, and *PiCesA4*) failed to prevent reduced cellulose accumulation in the cell wall, highlighting the essential role of *Pi*CESA2 in cell wall organization. A similar outcome was observed in the related oomycete species *Saprolegnia monoica*, where upregulation of all *CesA* genes was observed in response to glucan biosynthesis inhibitors, such as 2,6‐dichlorobenzonitrile or Congo Red (Fugelstad et al., 2009).

Alterations in *P. infestans* cell‐wall composition further support the antimicrobial activity of CP32. Across *in vitro* and *ex vivo* assays, and even in the absence of formulation or chemical modification, CP32 achieved levels of control comparable to those of commercial fungicides. The presence of multiple arginine residues in CP32 likely enhances its permeability into *P. infestans*, as revealed by staining with the membrane‐impermeant dye SYTOX Green and by the *in vitro* PAMPA‐BBB assay. Indeed, the peptide translocation across plasma membranes is often facilitated by arginine‐rich sequences or other guanidinium‐containing groups, which promote membrane interaction and uptake (Clark et al., 2021; Gan et al., 2021; Ji et al., 2024). Notably, sporangia exposed to CP32 for 3 d resumed growth upon peptide removal, supporting a fungistatic rather than cytotoxic mode of action—consistent with its lack of detectable toxicity toward plasma membranes—in agreement with previous observations (Ingham and Schneeberger, 2012; Velivelli et al., 2020).

Ultimately, the mode of action of CP32 appears to be unprecedented among commercial antifungal compounds, as it targets *Pi*CESA2, a protein that has not previously been exploited for plant protection. While inhibition of cellulose biosynthesis has previously been described as an effective antifungal strategy (Blum et al., 2010a, 2010b, 2012), the binding site engaged by CP32 within the TED motif of *Pi*CESA2 appears to be novel. This distinctive interaction differentiates CP32 from currently available compounds, which act on alternative targets, such as the cellulose synthase regulatory pathways (e.g., mandipropamid). Consequently, CP32 may serve as a valuable complementary agent when used in combination with existing fungicides, potentially broadening the spectrum of action and mitigating selection pressure on single targets. In addition, as a peptide‐based compound, CP32 offers opportunities for further optimization through formulation strategies aimed at improving stability, delivery, and field performance. Its biodegradable nature, together with the absence of phytotoxic effects on tomato and Arabidopsis tissues and its expected low environmental persistence, further supports its potential as a promising component of sustainable disease management strategies.

## MATERIALS AND METHODS

### Organisms and culture conditions

*Phytophthora infestans* isolate CBS 111345 was grown on pea‐agar medium (PAM; 12.5% (w/v) frozen peas and 1.2% (w/v) bacteriological agar) at 19°C. Sporangia suspensions were prepared from 11‐ to 15‐d‐old *P. infestans* mycelia grown in darkness on oatmeal‐agar medium (OMA; 6% (w/v) raw oat in distilled water). To collect sporangia, the plates were rinsed with a few milliliters of Pea Broth (PB; 12.5% (w/v) frozen peas in distilled water) or water. The suspension was then filtered through a 40‐μm cell strainer, counted using a Kova chamber (Kova Inc., Garden Grove, CA, USA), and adjusted to a final concentration of 1–2 × 10^4^ sporangia/mL.

*P. capsici* strain Hd11 and *P. ultimum* isolate (n. 724.94; CBS; Baarn, The Netherlands) were cultured on V8 Agar plates and PDA (Potato Dextrose Agar), respectively; the *P. viticola* monosporangial isolate was maintained on detached grapevine leaves (Massi et al., 2022).

*Solanum lycopersicum* cv. Marmande was grown under greenhouse conditions at 22°C, and leaves from 4–6‐week‐old plants were used for antimicrobial assays and leaf toxicity tests. To test the peptide effects and efficacy *in planta*, plants were grown at a short‐day photoperiod (12 h light/12 h darkness) for 2 weeks.

### RNA extraction and cloning protocols

Total RNA from *P. infestans* mycelia grown in liquid pea broth was extracted using the LiCl method (Vennapusa et al., 2020). Following the manufacturer's instructions, 1 μg of total RNA was retrotranscribed using the iScript™ gDNA Clear cDNA Synthesis Kit (Bio‐Rad Laboratories S.r.l.). *PiCesA2* was amplified with Q5 High‐Fidelity Polymerase (New England Biolabs, MA, USA) using the primers PiCesA2_GW_Fw and PiCesA2_GW_Rv under the following PCR conditions: 1× (98°C for 5 min), 35× (98°C for 30 s, 66°C for 30 s, 72°C for 15 s), and 1× (72°C for 5 min). PCR products were purified using NucleoSpin Plasmid (Macherey‐Nagel, Germany) and used for Gateway cloning (ThermoFisher Scientific, MA, USA).

### Yeast two‐hybrid peptide library screening

The Y187 yeast strain (*MATα*, *ura3‐52*, *his3‐200*, *ade2‐101*, *trp1‐901*, *leu2‐3*, *112*, *gal4Δ*, *met‐*, *gal80Δ*, *URA3::GAL1UAS‐GAL1TATA‐lacZ*) (Takara Bio USA, Inc., Japan) was transformed with pGBKT7–*Pi*CESA2 using the classical LiAc/SS carrier DNA/PEG methodology (Gietz and Schiestl, 2007) and plated on YSD − W. Transformed Y187 cells were grown in 50 mL of YSD − W to an OD_600_ of 0.8, and mated with the CYCLIC peptide library (Rosa et al., 2023). Cells were then plated on YSD − W − L − H − A + 10 mM 3‐AT (3‐amino‐1,2,4‐triazole) and incubated at 30°C for 7 d. Positive colonies were used as templates for PCR amplifications. CP‐encoding sequences were identified by Sanger sequencing (PlateSeq Kit PCR service, Eurofins Genomics, Germany). To test the interactions of the *Pi*CESA2 variants M1, M2, and M3 with CP32, pGBKT7 vectors harboring GAL4BD–PiCESA‐M_*x*_ fusions were introduced into the Y187 strain, which were later mated with the AH109 strain expressing the GAL4AD–CP32 fusion. Interaction tests were performed on auxotrophic media.

### Microwave‐assisted solid‐phase peptide synthesis

The cyclic peptides used in this work were synthesized using 2‐chlorotrityl chloride resin (0.3–0.6 mmol/g, 0.1 mmol scale). Coupling was performed with five equivalents of amino acids in DMF (0.2 M), using DIC (0.5 M in DMF), Oxyma Pure (1 M in DMF), and DIPEA (0.1 M) as reagents, at 50°C and 170 W. For arginine, a double coupling was used. Fmoc‐group deprotection was done with 20% (v/v) piperidine in DMF, at 25°C for 2 min, followed by 8 min at 50°C and 35 W. The peptide was cleaved from the resin by incubation with DCM/TFE/AcOH (8:1:1) for 2 h at room temperature. The solvent was evaporated under reduced pressure, water was added, and the suspension was freeze‐dried. The linear peptides were then dissolved in DMF (0.2 mM), and DIPEA was added until the solution reached pH 8. BOP (6 eq.) and HOBt (6 eq.) were then added, and the reaction was left under stirring at RT. After 6 h, BOP was added again, the pH was adjusted with DIPEA, and the reaction was stirred overnight. The mixture was then diluted with EtOAc and extracted with brine and saturated NaHCO_3_. The organic phase was then dried under reduced pressure, and the crude residue was reacted with the cleavage cocktail K TFA/phenol/H_2_O/thioanisole/EDT (82.5:5:5:5:2.5) and precipitated in Et_2_O after 3 h. The crude fraction was then dissolved in ACN/H_2_O with 0.1% TFA, purified on semi‐preparative RP‐HPLC, then lyophilized and stored at −15°C. A list of the so far synthesized cyclic peptides is shown in Table S5.

### Antimicrobial activity assays

#### *In vitro* efficacy assays

The antifungal activity of selected peptides was assessed at 200 μM in the presence of *P. infestans* using the 96‐well plate assay, as previously described (Marcianò and Toffolatti, 2023). Briefly, 50 μL of each peptide dilution was added to wells containing 50 μL of a 2 × 10^4^ sporangia/mL suspension. An untreated and a positive control with a commercial fungicide (mandipropamid, 10 mg/L) were also included. Growth inhibition was quantitatively measured by absorbance at 620 nm using a microplate reader after 72 h, and relative levels of growth inhibition were calculated accordingly.

Analysis of the dose response to CP32 was carried out, as described above, using 10‐fold decreasing peptide concentrations (200, 20, 2, 0.2, and 0 μM). Sporangia viability was assessed using the resazurin cell‐viability assay at decreasing peptide concentrations (200, 100, 50, 25, 12.5, 6.25, 3.12, and 0 μM), and absorbance was recorded at 600 nm after 72 h (Vega et al., 2012). EC_50_ and EC_99_ values were calculated as described by Ritz et al. (2015).

The activity of CP32 against related oomycete species was evaluated as follows: The activity against *P. capsici* was evaluated using the liquid medium (Potato Dextrose Broth (PDB)) assay in 96‐well plates inoculated with sporangia, while the activity against *P. ultimum* was assessed using the agar medium (PDA) assay inoculated with mycelium (Marcianò and Toffolatti, 2023). Mycelial growth was assessed after 3 d post‐inoculation (dpi).

#### *Ex vivo* efficacy assays

Three apical tomato leaflets were placed with their abaxial surfaces facing upwards in Petri dishes containing moistened filter papers. For infection, zoospore suspensions were prepared by resuspending sporangia in modified Petri's buffer (0.25 mM CaCl_2_, 1 mM MgSO_4_, 1 mM KH_2_PO_4_, 0.8 mM KCl) and stored at 4°C for 2 h to induce zoospore release. Different concentrations of CP32 (400, 200, 100, 50, 25 μM) were added to the zoospore suspension (1 × 10^4^ sporangia/mL) immediately prior to inoculation. Each leaflet was inoculated with three 10‐μL droplets of spore suspension. Three plates, each containing three leaflets, were used as replicas for each treatment. A negative control with sterile distilled water, instead of CP32, and a positive control with 10 mg/L of a commercial fungicide (Pergado SC; 250 g/L mandipropamid; Syngenta, Italy), were included. After inoculation, plates were stored in a growth chamber at 18°C ± 1°C (16 h light, 8 h dark). Disease severity was visually assessed at 5 dpi using a six‐class scale (0 = absence of necrosis, 1 = 0.1%–9%, 2 = 10%–24%, 3 = 25%–49%, 4 = 50%–74%, 5 = 75%–100%). The disease severity index (DSI) was calculated using the formula: DSI = 2.12*x* ^2^ + 9.41*x*, where *x* is the mean disease class value for each leaflet. DSI values were converted to Abbott's percentage protection index (PPI) (Abbott, 1925) and compared to the untreated control. A Kruskal−Wallis test followed by Fisher's least significant difference post‐hoc test, using the Kruskal function in the agricolae package, was performed to compare PPI values among treatments based on the average PPI from three plates (de Mendiburu, 2020).

*Ex vivo* assays on potato tubers were performed, as previously described (Ullah et al., 2026). Tubers were co‐inoculated with *P. infestans* sporangia after wounding. Increasing concentrations of CP32 peptide were used (50, 100, 200, and 400 μM). Three tubers per treatment were employed as biological replicas. Untreated (no CP32) and non‐infected (no *P. infestans*) controls were also included. Symptoms were visually assessed after 7 d post‐inoculation.

The activity against *P. viticola* was evaluated using the co‐inoculation assay, as previously described (Colombo et al., 2020). After inoculation, plates were stored in a growth chamber at 22°C ± 1°C with a photoperiod of 16 h light and 8 h dark. After the incubation period (7 d), disease severity was evaluated by assigning a class to each leaf disc based on the percentage of symptomatic leaf disc surface; PPI values were analyzed as described above.

#### *In planta* efficacy assay

Two pots containing six tomato plants were used as replicas for each treatment. Treatment was performed by spraying 1 ml of CP32 at desired concentrations (50 μM and 100 μM in water). Untreated (no CP32) and non‐infected (no *P. infestans*) controls sprayed with distilled water were also included. *P. infestans* sporangia propagated on leaves (Becktell et al., 2006) were collected with cold distilled water, and the concentration was adjusted to 2 × 10^4^ spores/mL. The spore suspension was kept at 4°C for 2 h to allow for zoospore release, and later 1 mL was sprayed on each replica pot. Plants were maintained in a growth cabinet (16 h light, 8 h dark, 25°C) and symptoms were scored 7 d post‐infection, using a five‐class scale (0 = absence of symptoms, 1 = 0.1%–1%, 2 = 2%–10%, 3 = 11%–25%, 4 = 26%–50%, 5 ≥ 51%) (EPPO, 2021). The disease severity index (DSI) was calculated using the formula: DSI = 6.57*x* ^2^ + 15.63*x* + 11.88, where *x* is the mean disease class value for each pot. DSI values were converted to Abbott's percentage protection index as described above.

### Quantification of crystalline cellulose deposition using confocal microscopy

Alterations in cell‐wall composition during short‐term adaptation to partially effective concentrations of CP32 were evaluated based on the level of cell‐wall regeneration of *P. infestans* protoplasts. *P. infestans* protoplasts were prepared by digesting 3‐d‐old mycelium grown in pea broth medium. Fungal material was resuspended in osmoticum (0.8 M mannitol, 20 mM KCl, 20 mM MES pH 5.7, 10 mM CaCl_2_) (Judelson et al., 1991) containing 15 mg/mL cellulase (CELLULYSIN®, Sigma Aldrich) and 5 mg/mL driselase (Driselase™ from Basidiomycetes sp., Sigma Aldrich) and shaken gently (60 rpm) at room temperature for 2 h. After digestion, protoplasts were filtered through Miracloth, harvested by centrifugation at 500 × *g* for 5 min, and washed twice in osmoticum before use.

Finally, protoplasts, adjusted to a final concentration of 1 × 10^7^, were resuspended in osmoticum containing CP32 at a sub‐optimal concentration (25 μM). An untreated sample was used as a control. To promote cell‐wall regeneration, cultures were gently vortexed for 30 s and further incubated at 18°C for 6 h, at which time cells were fixed using 4% (v/v) formaldehyde in PBS.

For cellulose quantification by means of confocal microscopy, cells were stained using a dye that is specific for crystalline cellulose (GFP‐CBM3A from *Clostridium thermocellum*, NZYTech). Before use, cells were rinsed three times for 5 min each in PBS solution to remove any residual traces of formaldehyde, before incubation with GFP‐CBM3A solution (20 μg/mL) for 45 min (Stroppa et al., 2023). Samples were observed using the ECLIPSE Ni‐E laser scanning confocal microscope (Nikon). The 488 nm laser line was used to excite GFP, and fluorescence was collected in the 500–550 nm emission windows.

Fluorescence intensity localized to the cystospore cell‐wall was measured using an automated image analysis script adapted from Miura and Sladoje (2020). In addition, the ratio of cell‐wall area to total cell area was calculated. All image analysis tasks were performed using the scikit‐image library in Python. An ANOVA was conducted to compare the values across different treatments.

### *CesAs* gene expression analysis

To examine transcript levels of all four *PiCesA* genes at different developmental stages, protoplast‐derived structures (prepared as previously described) were sampled 3 and 6 h after inoculation with CP32 (25 μM). In addition, samples of mycelia (3 d old), zoospores, and untreated protoplasts (at 0, 3, and 6 h) were also collected. Total RNA was extracted using the Arcturus PicoPure kit (ThermoFisher Scientific, USA). After DNase digestion, first‐strand cDNA was synthesized, using the iScript gDNA Clear cDNA synthesis kit (Bio‐Rad, USA). RT‐qPCR was performed on the PCR system (CFX96 Real‐Time System, Bio‐Rad) using the iTaq Universal SYBR Green Supermix (Bio‐Rad, USA) together with the cDNA template and suitable primers for amplification (Grenville‐Briggs et al., 2008; see also Table S5). The relative expression level of the target gene was calculated using the 2^−ΔΔCt^ method, and *ACTIN* (GenBank ID: M59715.1) was used as the reference. The expression of each *PiCesA* gene in the mycelial cDNA sample was normalized to a value of 1.0. Means and standard deviations were calculated based on data from three replicates.

### Biochemical characterization of the cell‐wall composition of *P. infestans*

The absolute quantification of changes in cell‐wall composition during long‐term adaptation to sub‐optimal concentrations of CP32 was assessed in mycelial samples of *P. infestans*. Mycelial plugs (5 mm in diameter) were inoculated into 9‐cm Petri dishes containing 15 mL of modified Plich's medium without yeast extract (Kamoun et al., 1993). After 3 d of incubation, the medium was replaced with fresh Plich's medium containing 25 μM CP32, together with a negative control without the peptide, and incubated for an additional 3 d. Following the incubation period, mycelia were washed three times with distilled water, and mycelial plugs were excised to prevent contamination with foreign polysaccharides.

Dried mycelial samples (1 mg) were treated with 72% H_2_SO_4_ for 2 h at room temperature, followed by heating at 100°C for another 2 h. The hydrolyzed samples were then diluted and filtered through 0.2‐μm nylon syringe filters before injection. Monosaccharide composition was analyzed using High‐Performance Anion‐Exchange Chromatography with Pulsed Amperometric Detection (HPAEC‐PAD) on a Dionex IC6000 system, controlled by Chromeleon software (version 7.3). Separation was performed on a CarboPac PA1 column (4 × 250 mm) at 20°C with a flow rate of 1 mL/min. The eluents used were water (Eluent A), 200 mM NaOH (Eluent B), and 200 mM NaOH in 170 mM NaOAc (Eluent C). Monosaccharides were identified and quantified by comparing retention times and peak areas with glucose standards (Sigma‐Aldrich) at concentrations ranging from 0.005 to 0.1 g/L. For glycosidic linkage analysis, the Alcohol‐Insoluble Fraction (AIF) was prepared from dried mycelia, and partially methylated alditol acetate (PMAA) derivatives were prepared following established protocols (Mélida et al., 2013) and analyzed by gas chromatography (GC) coupled with an electron‐impact mass spectrometer (EI‐MS). The PMAA derivatives were separated on an SP‐2380 capillary column, and their mass spectra were compared with reference derivatives (cellulose, curdlan, and laminarin) and available data to quantify relative changes in cell wall polysaccharide composition.

### *In silico* predictions of the peptide‐target interaction site

The glycosyltransferase (GT) domain was predicted using DeepMind AlphaFold2 (Jumper et al., 2021), and the input sequence contained residues 644–778 (VRDNAVAFVQTPQYFRDCIQLQIGDPMGHRNATFYDAIQTGQDGYDCASFAGTNAMFRREALDSIGGIQYGSLTEDCYTGQVLCSMGWKAQYFRKDFEGEPSERIRLAEGLIPDSVAGSLAQRKRWAKGNFQIAL). As part of the AlphaFold2 pipeline, the HHsuite (Steinegger et al., 2019) identified templates from the Protein Data Bank (PDB) (Berman et al., 2000) with the following entries: 5EJ1 (chain A), 4P00 (chain A), 7LBY (chain A), 7D5K (chain A), 8G27 (chain D), 6WLB (chain B), 8DQK (chain A), 7CK3 (chain A), 6YV9 (chain A), 7SP7 (chain A), 7SPA (chain A), 7CK1 (chains A and B), 7CK2 (chain A), 7SP6 (chain A), 7SP8 (chain A), 7WJO (chain A), 7×06 (chain B), and 7WJM (chain B).

The model, which, after 12 cycles, achieved the highest predicted local distance difference test (pLDDT) score of 93.7 and the highest predicted template modeling (pTM) score of 0.833, was selected for further relaxation, performed using the gradient descent method with the Amber (Hornak et al., 2006) force field. Additional refinement of the model was conducted using the Protein Preparation Wizard tool (Madhavi Sastry et al., 2013) from the Schrödinger Maestro suite version 13.9.138, release 2024‐1 (Maestro, Schrödinger, LLC, 2024). This process included the assignment of the proper protonation states for ionizable groups at pH 7 ± 1, optimizing the orientations of hydroxyl and amide moieties to enhance the hydrogen bond network, and minimizing energy (using the OPLS (Lu et al., 2021) force field) to resolve steric clashes and other unfavorable interactions.

A second model was predicted using as input the aa sequence of *Pi*CESA2 in the range 197–1,026. This model achieved pLDDT and pTM scores of 83.9 and 0.815, respectively. The model was then inserted into a phospholipid bilayer membrane composed of 50% 1‐palmitoyl‐2‐oleoyl‐sn‐glycero‐3‐phosphoethanolamine (POPE) and 50% 1‐palmitoyl‐2‐oleoyl‐sn‐glycero‐3‐phosphocholine (POPC), using the CHARMM‐GUI (Jo et al., 2008) online tool with the support of the Positioning of Proteins in Membranes (PPM) webserver, version 2.0 (Lomize et al., 2012).

As β‐alanine was absent from the native peptide identified by the screening process, it was designated as position zero (0), preceding the eight amino‐acid residues derived from the “original” sequence tested *in vivo*.

The cyclic peptide CP32, cyclo (βAla‐Gly1‐Arg2‐Leu3‐Arg4‐Trp5‐Leu6‐Arg7‐Val8), was initially predicted in an open conformation using PEP‐FOLD3 (Shen et al., 2014) with the input sequence GRLRWLRV. The model closest to a cyclic conformation was selected for further modification using the open‐source version of PyMOL (Schrödinger, LLC. The PyMOL Molecular Graphics System, Version 2.6). The β‐alanine residue was manually modeled starting from an α‐alanine residue and used to link Gly1 to Val8, closing the peptide into a cyclic conformation. The geometries and protonation states of the ligand were optimized using the LigPrep (Madhavi Sastry et al., 2013) tool.

For docking, a cubic grid box with an edge length of 76 Å was designed to contain the entire predicted model of the *Pi*CESA2 catalytic domain, allowing for a blind docking procedure using the Schrödinger Maestro suite with GlideXP (Friesner et al., 2006) and flexible ligand sampling.

Structure and sequence similarity analyses of the *Pi*CESA2 catalytic domain were conducted against the RCSB PDB100 database subset using the Foldseek (van Kempen et al., 2024) webserver. All molecular structure images were created using the open‐source version of PyMOL.

## CONFLICTS OF INTEREST

The following authors, P.P., S.M., S.R., C.M., A.T., S.Pe., L.F., S.L.T., D.M., V.B., V.S., and S.O. are listed as inventors on the following patent application, which covers a family of peptides, among which CP32 is included. The remaining authors declare no conflicts of interest.

WO2025093718 ANTIMICROBIAL PEPTIDES FOR THE CONTAINMENT OF OOMYCETES IN AGRICULTURE: P. Pesaresi, S. Masiero, S. Rosa, C. Mizzotti, A. Tagliani, S. Pellegrino, L. Feni, S.L. Toffolatti, D. Marcianò, V. Bulone, V. Srivastava, S. Ongeri, L. Radal.

## AUTHOR CONTRIBUTIONS

D.M., S.R., P.P., and S.M. designed the experiments and analyzed data. S.R., E.M.F., and C.M. screened the peptide library, and N.G.C. performed the Y2H assays. D.M. and E.M.F. evaluated the cellulose accumulation, and with S.L.T. evaluated the antimicrobial activity of CP32. F.B. and C.C. ran the docking analysis. G.R. assisted with the MTT assays and S.Pr. with the PAMPA assays. L.F., S.Pe., and S.O. synthesized part of the peptide used in the manuscript. A.T. and C.B. tested the mutated versions of the cellulose synthase and CP32. L.T., L.J.G., and R.R. performed the pull‐down and MST analysis. V.S., L.K., S.F.U., D.M., and V.B. quantified the cell wall composition in the presence of CP32. S.R., D.M., S.M., and P.P. wrote the initial draft, which was sent to all authors, and L.J.G. verified the appropriateness of the manuscript's language. All authors have read and approved the contents of this paper.

## Supporting information

Additional Supporting Information may be found online in the supporting information tab for this article: http://onlinelibrary.wiley.com/doi/10.1111/jipb.70343/suppinfo

**Figure S1.** CP32 antimicrobial effects on *Phytophthora infestans* infection in potato tuber sections
**Figure S2.** Multiple sequence alignment (MSA) of the cellulose synthase (CESA) catalytic domains
**Figure S3.** Confocal micrographs of *P. infestans* labeled with GFP‐CBM3A
**Figure S4.** CP32‐FITC localizes at the plasma membrane interface
**Figure S5.** Interaction of CP32 with *Pi*CESAs in the yeast two‐hybrid assay
**Figure S6.** Biochemical characterization of the *Pi*CESA2‐CP32 interaction
**Figure S7.** Comparison of the *Cereibacter sphaeroide*s cellulose synthase GT domain structure with that of the *P. infestans* cellulose synthase A2 GT domain model
**Figure S8.** CP32 cyclic peptide docking poses on the *Pi*CESA2 GT domain
**Figure S9.** Alanine scans reveal that residues R2 and W5 of CP32 are essential for its interaction with *Pi*CESA2
**Figure S10.** SYTOX‐Green staining of *P. infestans* hyphae treated or not with CP32
**Figure S11.** Effects of CP32 on the germination of *P. infestans* sporangia
**Figure S12.** Assessment of CP32's biological activity on microorganisms and human cells
**Figure S13.** Assessment of CP32 biological activity on tomato leaves and on hypocotyl growth in Arabidopsis
**Figure S14.** Assessment of CP32 treatment on tomato plant growth
**Table S1.** List of peptides that specifically interact with *Pi*CESA2, identified by Y2H screening
**Table S2.** Disease severity assessment for the *in vivo* assay performed on tomato plants
**Table S3.** Spectrum of action of CP32 against other phytopathogenic oomycete species (*P. capsici*, *P. ultimum* and *P. viticola*)
**Table S4.** PAMPA‐BBB permeability classification of reference compounds used as a training set
**Table S5.** List of the peptides synthesized and used in this work
**Table S6.** List of primer sequences used for gene cloning and gene expression analyses
