# Supplementary material for: CP32, a novel anti‐oomycete cyclic peptide, interferes with cell‐wall deposition in Phytophthora infestans
Source: J Integr Plant Biol. 2026 Jul 16;68(11):4013–29. doi: 10.1111/jipb.70343 (PMC13725601; doi:10.1111/jipb.70343)
Supplement: Supplementary file 1 [file JIPB-68-4013-s001.docx]

**CP32, a novel anti-oomycete cyclic peptide, interferes with cell-wall deposition in *Phytophthora infestans***

Demetrio Marcianò^1†^, Stefano Rosa^1†^, Elena Marone Fassolo^1^, Chiara Bertaso^1^, Andrea Tagliani^1^, Nina Gauri Capra^1^, Federico Ballabio^1^, Chiara Mizzotti^1^, Giulia Rizzi^1^, Sadia Fida Ullah^2^, Lisa Kappel^2^, Lucia Feni^3^, Luca Tadini^1^, Louise Jane Gourlay^1^, Rosaria Russo^4^, Vincent Bulone^5^, Sabrina Pricl^6,7^, Sandrine Ongeri^8^, Sara Pellegrino^3^, Carlo Camilloni^1^, Silvia Laura Toffolatti^9^, Vaibhav Srivastava^2^, Paolo Pesaresi^1^, Simona Masiero^1^*

^1^Dipartimento di Bioscienze Università degli Studi di Milano, Milano 20133, Italy

^2^Division of Glycoscience, Department of Chemistry, School of Engineering Sciences in Chemistry, Biotechnology and Health, Royal Institute of Technology (KTH), SE 106 91 Stockholm, Sweden

^3^Dipartimento di Scienze Farmaceutiche, Università degli Studi di Milano, Milano 20133, Italy

^4^Dipartimento di Fisiopatologia Medico-Chirurgica e dei Trapianti, Università degli Studi di Milano Milano 20100, Italy

^5^College of Medicine and Public Health, Flinders University, Bedford Park Campus, SA 5042, Australia

^6^Molecular Biology and Nanotechnology Laboratory (MolBNL@UniTS), DEA, University of Trieste, Trieste 34127, Italy

^7^Department of General Biophysics, Faculty of Biology and Environmental Protection, University of Lodz, 90-236 Łódź, Poland

^8^Université Paris-Saclay, CNRS, BioCIS, Bat. Henri Moissan, 91400 Orsay, France

^9^Dipartimento di Scienze Agrarie e Ambientali - Produzione, Territorio e Agroenergia, Università degli Studi di Milano, 20133 Milano 20133, Italy

^†^These authors contributed equally

*Correspondence: Simona Masiero (simona.masiero@unimi.it)

**SUPPLEMENTARY METHODS**

***CesAs* gene expression analysis**

To examine transcript levels of all four *PiCesA* genes at different developmental stages, protoplast-derived structures (prepared as previously described) were sampled 3 and 6 h after inoculation with CP32 (25 µM). In addition, samples of mycelia (3 days old), zoospores and untreated protoplasts (at 0, 3, and 6 h) were also collected. Total RNA was extracted using the Arcturus PicoPure kit (Thermo Fisher Scientific, USA). After DNase digestion, first-strand cDNA was synthesized, using the iScript gDNA Clear cDNA synthesis kit (Bio-Rad, USA). qRT-PCR was performed on the PCR system (CFX96 Real-Time System, Bio-Rad) using the iTaq Universal SYBR Green Supermix (Bio-Rad, USA) together with the cDNA template and suitable primers for amplification (Grenville-Briggs et al., 2008; see also Table S5). The relative expression level of each target gene was calculated using the 2^–ΔΔCt^ method, with *actin* (GenBank ID: M59715.1) as the reference. The expression of each *PiCesA* gene in the mycelial cDNA sample was normalized to a value of 1.0. Means and standard deviations were calculated based on data from three replicates.

**Biochemical characterization of CP32-PiCESA2 interaction via pull-down and MST assays.**

The PiCESA2 (318–770) expression construct was generated by gene synthesis and cloning (Officinae Bio, Venice, Italy) and cloned into the pET14b vector to produce an N‑terminal His_6_-tagged protein. The resulting plasmid was transformed into *Escherichia coli* Rosetta (DE3) pRARE pLysS competent cells using standard procedures. Transformants were selected on LB agar supplemented with ampicillin (100 µg/mL) and chloramphenicol (50 µg/mL). Single colonies were grown overnight at 37°C and used to inoculate three 1.5 L LB cultures containing the same antibiotics.

Protein expression was induced at OD₆₀₀ = 1.5 by addition of 0.5 mM IPTG, followed by overnight incubation at 20°C with shaking. Cells were harvested by centrifugation and resuspended in lysis buffer (10 mM Tris‑HCl pH 7.5, 300 mM NaCl) supplemented with DNase, RNase, lysozyme, and cOmplete™ Protease Inhibitor tablets (Roche). After incubation at 37°C for 30 min, cells were lysed by sonication, and the lysate was clarified by centrifugation at 12,000 × g for 45 min at 4°C.

The soluble fraction was incubated with Ni-NTA affinity resin (Merck Millipore) for 2 h at 4°C. The resin was washed with buffer containing 40 mM imidazole, and bound proteins were eluted using 300 mM imidazole. Eluted fractions containing recombinant PiCESA2 (318–770) were used for subsequent experiments.

For pull‑down assays, FITC-labeled CP32 peptide (CASLO ApS) was incubated with increasing amounts of purified His_6_::*Pi*CESA2 (318–770) protein in pull‑down buffer supplemented with cOmplete™ Protease Inhibitor Cocktail (Roche). Samples were incubated with Ni‑NTA agarose resin at 4°C, washed four times, and bound proteins were eluted in sample buffer. Eluted proteins were resolved by 15% SDS-PAGE, transferred onto nitrocellulose membranes (Amersham), and analyzed by immunoblotting using anti‑His and anti‑FITC antibodies (Thermo Fisher Scientific).

For microscale thermophoresis (MST) experiments, IMAC-purified *Pi*CESA2 (318–770) was concentrated to 500 µL and loaded on a Superdex 200 10/300 Increase size exclusion chromatography (SEC) column (Cytiva), pre-equilibrated in 1× PBS at a flow rate of 0.5 mL/min, using an AKTAPure FPLC instrument (Cytiva). 1 mL fractions were collected and *Pi*CESA2-containing fractions, were used directly for microscale thermophoresis (MST) experiments. 7.6 µM *Pi*CESA2, eluted from the SEC column was diluted using Monolith PBS-T (PBS containing 0.05% (v/v) Tween-20) buffer to 200 nM, and fluorescently labeled, using the His-Tag Labeling Kit RED-tris-NTA 2nd Generation (NanoTemper Technologies), following the manufacturer’s high affinity protocol. The CP was diluted to 25 mM with PBS-T.

MST experiments were carried out at 25°C (excitation power 5%; MST power 40%), using the Red LED (600–650 nm excitation range), at 16 different ligand concentrations (0.8 μM to 12.5 mM) and a NanoTemper® Monolith NT.115 instrument. 10 μL of the highest ligand concentration (25 mM) were added to the first tube, while 10 μL of PBS-T were added to the other 15 tubes. Serial, 1:1 dilutions of the ligand were performed, mixing thoroughly. Finally, 10 μL fluorescently-labeled *Pi*CESA2 (318–770) (100 nM) were added to each tube, and mixed by pipetting, before loading into standard Monolith capillaries. MST curves, corresponding to temperature-induced fluorescence intensity changes, were recorded over time at each ligand concentration. A dose-response curve, plotting the change in normalized (baseline-corrected) temperature-related fluorescence intensity changes (Δ F_norm_), expressed in parts per thousand (‰), as a function of the logarithm of the ligand concentration, was generated. The curve was used to estimate the binding affinity by applying the law of mass action.

**Heterologous expression of *Pi*CESA2 in *S. cerevisiae* and activity assay**

The *PiCesA2* gene (XM_002902461.1) was amplified using the primers Cln_NGFP_PiCesA2_FW and Cln_NGFP_PiCesA2_RV and cloned into the pDDGFP-N-His_8_-yEGFP vector. The *S. cerevisiae* strain YEF473A (Bi and Pringle, 1996) was used for protein expression.

The transformant showing the highest protein expression level was selected for recombinant protein production using 2% (w/v) galactose, 0.04% (w/v) histidine and 2.5% (v/v) DMSO for induction, and expression was induced for 22 h at 250 rpm and 30°C during logarithmic growth (Drew et al., 2008). His_8_-yEGFP::PiCESA2 was extracted from the microsomal fraction using 0.5% (w/v) digitonin (Drew et al., 2008; Pang et al., 2020) and purified using immobilized-metal-affinity chromatography (IMAC) in a buffer system containing 1× PBS (pH 8.0), 150 mM NaCl, 0.05% (w/v) digitonin, 10% (v/v) glycerol and increasing imidazole concentrations. Fractions containing the protein were then concentrated using Amicon Ultra centrifugal filters (cutoff 10 kDa) (Merck Millipore, Cork, Ireland). Buffer exchange was performed against 1×PBS, pH 7.4 and the integrity of the product was verified using in-gel fluorescence.

Reaction mixtures for activity determination contained 85 ng mL^−1^ purified His_8_-yEGFP-PiCESA2 prepared in 10 mM PIPES buffer (pH 6.0), 10 mM MgCl2, 0–1.5 mM UDP-Glc as substrate and 2 mM Glc as acceptor in a total volume of 25 μL and were incubated for 2 h at 22°C. 0 µM, 100 µM and 200 µM CP32 was added 30 min prior to the addition of UDP-Glc. Reactions were stopped by adding 25 μL of the UDP-Glo reagent (Promega) and developed for 1 h at 25°C. The plates were then transferred into a plate reader (Clariostar). All experiments were performed in triplicate.

***In vivo* localization of CP32-FITC**

*P. infestans* sporangia suspension (1–2 × 10⁴ sporangia/mL) was incubated overnight in Pea Broth at 19°C to promote germination. Germling suspensions (1–2 mL) were incubated with 2.5 µM FM 4-64 (Thermo Fisher Scientific) for 10 minutes on ice and in the dark. Samples were centrifuged at 700 × g for 5 minutes, and the pellet was resuspended in an equal volume of fresh Pea Broth. Treated samples were prepared by adding CP32-FITC (CASLO ApS) at 16 µM immediately before spotting samples on glass slides and covering with coverslips. Samples were examined using an ECLIPSE Ni-E laser scanning confocal microscope (Nikon). FITC-CP32 and FM 4-64 were excited at 488 nm and emission collected at 510–530 nm and 620–660 nm, respectively.

**Evaluation of CP32 membrane permeability and its effects on non-target organisms**

The passive permeability of CP32, CSA, CSD and EnB, together with an additional set of 27 compounds, was evaluated using PAMPA-BBB as described by Di et al. (2003), following an established protocol (Estrada-Valencia et al., 2019; Campora et al., 2021; Rosa et al., 2023). A semi-automated pipetting device (BenchSmart 96, Mettler Toledo) was used for liquid handling, while a microplate spectrophotometer (SpectraMax Plus 384, Molecular Devices) was employed for UV absorbance measurements. Porcine brain lipid (PBL, catalog number 141101C) was sourced from Avanti Polar Lipids, and Millex filter units (PVDF membrane, 0.45 μm pore size) were purchased from Millipore. All commercial chemicals and reagents were obtained from Sigma-Aldrich. The acceptor microplate (PTFE, Millipore) was prepared by adding 300 μL of PBS/DMSO (95:5, pH 7.4) to each well, while the donor microplate membrane (PVDF, 0.45 μm pore size, Millipore) was coated with 5 μL of PBL dissolved in dodecane (20 mg/L). Each compound was dissolved in DMSO, diluted with PBS/DMSO (95:5, pH 7.4) to a final concentration of 40–100 μM, filtered through a Millex filter, and added to the donor microplate wells (200 μL per well). The donor plate was placed on top of the acceptor plate to bring the artificial membrane into contact with the buffer below. The system was then sealed with damp paper towels to prevent evaporation, and incubated undisturbed for 18 h at 25°C. After incubation, the donor microplate was removed, and UV-visible spectrophotometry was used to measure the amounts of the tested compounds in the donor and acceptor wells. Each sample was measured at five different wavelengths in four replicate wells across three independent runs. The data are presented as mean values with standard deviation. The permeability coefficients (P_e,exp_, cm/s) were calculated using the equation: P_e,exp_ = {−V_d_V_a_/[(V_d_ + V_a_)A_t_]ln(1) − d_a_/d_eq_}, where V_d_ and V_a_ are the volumes of the donor and acceptor wells, respectively, A is the area of the artificial membrane, t is the permeation time, and d_a_ and d_eq_ are the absorbances measured in the acceptor well and the theoretical equilibrium absorbance, respectively. After the test was completed for all compounds, the integrity of the lipid membrane was assessed using Lucifer Yellow (Sigma-Aldrich), a fluorescent molecule with limited permeability, to confirm the uniformity of the membrane, as described in the Millipore protocol (PC1545EN00) https://www.sigmaaldrich.com/technical-documents/protocols/biology/membrane-integrity-test-for-lipid-pampa-artificial-membranes.html.

A *P. infestans* sporangia suspension (1–2 × 10⁴ sporangia/mL) was inoculated into pea broth, and cultured for two days under continuous agitation. The recovered mycelia were then treated with 10 nM SYTOX Green (ThermoFisher Scientific) and incubated in the dark at room temperature for 5 min. After this incubation, CP32 was added at its EC_50_ concentration. Fluorescence microscopy was carried out using standard fluorescein settings (excitation: 495 nm, emission: 517 nm) within a time frame of 30 to 90 min after peptide incubation for each treatment.

In addition, a growth restoration assay was performed (Figure S7). A *P. infestans* sporangia suspension (1–2 × 10⁴ sporangia/mL) was grown in pea broth supplemented with either CP32 (EC_50_ concentration) or water (negative control). Cultures were observed at 3 dpi using a Zeiss Axiophot D1 microscope equipped with differential interference contrast (DIC) optics. Images were captured with an Axiocam MRc5 camera (Zeiss) using the Axiovision software (version 4.1). CP32-treated samples were centrifuged at 700 rcf for 5 minutes, washed, and resuspended in fresh pea broth. The cultures were then re-examined under the microscope following the protocol described by Velivelli et al. (2020).

The biological activity of CP32 was also assessed on non-target bacterial species (*Escherichia coli* strain DH10β and *Agrobacterium tumefaciens* strain GV3101) and on the eukaryotic model organism *Saccharomyces cerevisiae* (strain AH109) by evaluating cellular growth in liquid cultures. Overnight pre-cultures were diluted to an OD₆₀₀ of 0.1 in sterile LB or YPDA medium for bacteria and yeast, respectively. CP32 was then added to the corresponding liquid medium at EC_99_ concentrations determined against *P. infestans*. Microorganisms were incubated at 28°C (*A. tumefaciens* and *S. cerevisiae*) or 37°C (*E. coli*), and cell population density was monitored spectrophotometrically at OD₆₀₀ over 24 h. Each assay was performed in triplicate. The potential phytotoxicity of the selected peptides at their EC_99_ concentrations was assessed by measuring the photosynthetic performance of *Solanum lycopersicum* cv. Marmande leaves. Maximum quantum yield (FV/FM) and effective quantum yield [Y(II)] of photosystem II were recorded after 7 days of incubation at room temperature using an Imaging PAM system (WALZ), as previously described (Perreault et al., 2010; Colombo et al., 2020). Glyphosate (1.8 g/L) was used as a positive control, while mock treatments were performed with water.

To assess the role of CP32 in primary cell-wall formation, Arabidopsis seeds were surface-sterilized, suspended in sterile distilled water, and plated on a 0.7% (w/v) agar medium with half-strength MS salts (Sigma-Aldrich) without sucrose. Treatments were added before pouring the media at a final concentration of either 20 µM, or 50 µM CP32, together with 1% (v/v) DMSO and 5 nM Isoxaben (Sigma-Aldrich cod. 36138) (Broad et al., 2024). For each treatment 35 replicas were considered. Plated seeds were cold-treated at 4°C for 3 days in the dark. Arabidopsis seeds were then exposed to a 6-hour white fluorescent light (150 µmol/m²/s) and vertically grown in complete darkness at 22°C. Seedlings were photographed using a Leica stereo microscope. Hypocotyl length was measured using ImageJ (W.S. Rasband; National Institutes of Health).

**References**

**Bi E, Pringle JR** (1996) ZDS1 and ZDS2, genes whose products may regulate Cdc42p in Saccharomyces cerevisiae. Mol Cell Biol **16**: 5264–5275

**Broad RC, Ogden M, Dutta A, Dracatos PM, Whelan J, Persson S, Khan GA** (2024) The fnr-like mutants confer isoxaben tolerance by initiating mitochondrial retrograde signalling. Plant Biotechnol J **22**: 3000–3011

**Campora M, Canale C, Gatta E, Tasso B, Laurini E, Relini A, Pricl S, Catto M, Tonelli M** (2021) Multitarget Biological Profiling of New Naphthoquinone and Anthraquinone-Based Derivatives for the Treatment of Alzheimer’s Disease. ACS Chem Neurosci **12**: 447–461

**Colombo M, Masiero S, Rosa S, Caporali E, Toffolatti SL, Mizzotti C, Tadini L, Rossi F, Pellegrino S, Musetti R, et al** (2020) NoPv1: a synthetic antimicrobial peptide aptamer targeting the causal agents of grapevine downy mildew and potato late blight. Sci Rep. doi: 10.1038/s41598-020-73027-x

**Di L, Kerns EH, Fan K, McConnell OJ, Carter GT** (2003) High throughput artificial membrane permeability assay for blood-brain barrier. Eur J Med Chem **38**: 223–232

**Drew D, Newstead S, Sonoda Y, Kim H, von Heijne G, Iwata S** (2008) GFP-based optimization scheme for the overexpression and purification of eukaryotic membrane proteins in Saccharomyces cerevisiae. Nat Protoc **3**: 784–798

**Estrada-Valencia M, Herrera-Arozamena C, Pérez C, Viña D, Morales-García JA, Pérez-Castillo A, Ramos E, Romero A, Laurini E, Pricl S, et al** (2019) New flavonoid - N,N-dibenzyl(N-methyl)amine hybrids: Multi-target-directed agents for Alzheimer´s disease endowed with neurogenic properties. J Enzyme Inhib Med Chem **34**: 712–727

**Grenville-Briggs LJ, Anderson VL, Fugelstad J, Avrova AO, Bouzenzana J, Williams A, Wawra S, Whisson SC, Birch PRJ, Bulone V, et al** (2008) Cellulose synthesis in Phytophthora infestans is required for normal appressorium formation and successful infection of potato. Plant Cell **20**: 720–738

**Perreault F, Oukarroum A, Pirastru L, Sirois L, Gerson Matias W, Popovic R** (2010) Evaluation of Copper Oxide Nanoparticles Toxicity Using Chlorophyll a Fluorescence Imaging in Lemna gibba. J Bot **2010**: 763142

**Rosa S, Tagliani A, Bertaso C, Tadini L, Visentin C, Gourlay LJ, Pricl S, Feni L, Pellegrino S, Pesaresi P, et al** (2023) The cyclic peptide G4CP2 enables the modulation of galactose metabolism in yeast by interfering with GAL4 transcriptional activity. Front Mol Biosci **10**: 1017757

**Velivelli SLS, Czymmek KJ, Li H, Shaw JB, Buchko GW, Shah DM** (2020) Antifungal symbiotic peptide NCR044 exhibits unique structure and multifaceted mechanisms of action that confer plant protection. Proc Natl Acad Sci U S A **117**: 16043–16054

**Figure S1. CP32 antimicrobial effects on *Phytophthora infestans* infection in potato tuber sections.**

Potato tuber slices were wounded and co‑inoculated with *P. infestans* sporangia and increasing concentrations of the CP32 peptide (0–400 µM). At seven days post‑inoculation, clear late blight symptoms were evident in the control sample (CP32 0 µM), whereas treatment with CP32 at concentrations above 100 µM completely inhibited visible disease development. Data represent three independent biological replicates (*n* = 3). Scale bar: 5 mm.

**Figure S2. Multiple sequence alignment (MSA) of the cellulose synthase (CESA) catalytic domains.**

MSAs were generated using Clustal Omega and visualized with Jalview (v. 2.11.5.1) using the conservation color scheme, in which darker shading indicates higher sequence conservation (Waterhouse et al., 2009). The alignment includes only the CESA catalytic domain and corresponds to residues 640–778 of *Pi*CESA2, which was used as bait in Y2H assays. Accession numbers used are: *Pi*CESA2 (D0NDY8), *Pv*CESA2 (D4N2S6), *Pu*CESA2 (K3WCB5; UniProt); *Pc*CESA2 (Phyca11_509421; *P. capsici* genome: genome.jgi.doe.gov/Phyca11/Phyca11.home.html), *Pi*CESA1 (D0NDY7), *Pi*CESA3 (D0NUK6), *Pi*CESA4 (D0NUJ1). **(A)** Alignment of *Phytophthora infestans* (*Pi*CESA1–*Pi*CESA4) catalytic domains, **(B)** *Phytophthora infestans* CESA2 sequence alignment: *Phytophthora capsici*, *Plasmopara viticola* and *Pythium ultimum*.

**Figure S3. Confocal micrographs of *P. infestans* labeled with GFP-CBM3A.**

**(A**–**D)** *De novo* cell-wall deposition in germinating sporangia (*n* = 15). **(E**–**H)** Encysted zoospores (*n* = 22). Note that CP32 treatment reduces cellulose deposition. Scale bar: 5 μm.

**Figure S4. CP32-FITC localizes at the plasma membrane interface.**

**(A**–**C)** Fluorescence confocal microscopy images of sporangial germ tubes treated with FITC‑labeled CP32 peptides **(A)** and stained with the membrane dye FM4‑64 **(B)**. Green fluorescence indicates the intracellular localization of CP32‑FITC, while red fluorescence corresponds to the cytoplasmic membrane labeled with FM4-64. The fluorescence channels were merged and overlaid onto the corresponding bright‑field images **(C)**. CP32-FITC signal was detected predominantly at the plasma membrane and displayed a punctate distribution, accumulating at specific discrete sites along the membrane (*n* = 10 germ tubes). This localization pattern is consistent with the predicted localization of cellulose synthase (CESA) enzymes organized in membrane‑associated multimeric complexes. Scale bar: 5 µm.

**Figure S5. Interaction of CP32 with *Pi*CESA in the yeast two-hybrid assay.**

CP32 specifically interacts with the catalytic domain of *Pi*CESA2, but not with *Pi*CESA1 and 3. The PiCesA4 catalytic domain, cloned into the pGBKT7 vector, exhibited weak autoactivation. CP32 did not interact with any of the four tested plant CesA catalytic domains (*St*CESAlike E1, *Sl*CESAlike E1, *St*CESA2, *Sl*CESA4).

**Figure S6. Biochemical characterization of the *Pi*CESA2-CP32 interaction**.

**(A)** In gel fluorescence of proteins purified by IMAC. Yeast cells were transformed with either the empty expression vector (GFP) or the vector carrying the *Pi*CesA2 gene fused to His_8_­-yEGFP. The position of full-length His_8_-yEGFP::CESA2 is indicated by the plus symbol. MW markers are indicated in kDa. **(B)** Transferase activity of recombinant *Pi*CESA2 from yeast. UDP released in the presence of increasing concentrations of CP32 (0–200 µM) was quantified using the UDP-Glo assay (Promega) and µM UDP mg^−1^ protein h^−1^ was calculated from a standard curve generated using a dilution series of UDP. Data from two independent experiments with standard deviations are shown. Statistical significance was determined using Student’s *t*-test (*P* < 0.05). **(C)** The interaction between FITC-labeled CP32 and His_6_::*Pi*CESA2 (residues 318–770) was assessed using a pull-down assay. Co-purification was performed with Ni-NTA agarose resin by incubating 100 µM CP32-FITC peptide with increasing amounts (0, 10 µg, and 50 µg) of purified His_6_::*Pi*CESA2 protein. The Ni-NTA-bound fraction (pull-down) was collected to evaluate the interaction, alongside the supernatant (unbound) fraction. Detection of the protein and peptide was carried out using antibodies against the His and FITC tags, respectively. **(D)** MST binding curve of the interaction between CP32 and *Pi*CESA2. The binding between CP32 and RED-Tris-NTA-labeled-*Pi*CESA2 was monitored by microscale thermophoresis. Increasing concentrations of CP32 (0.8 μM–12.5 mM final concentration) were titrated into fluorescently-labeled *Pi*CESA2 (100 nM) and the resulting MST traces were recorded over time. The resulting dose-response curve shows the change in normalized (baseline-corrected) temperature-related fluorescence intensity changes (ΔFnorm), expressed in parts per thousand (‰), as a function of the logarithm of the ligand concentration. The dissociation constant was estimated by fitting the curve to the law of mass action, however, even at 12.5 mM CP32, binding saturation was not achieved.

**Figure S7. Comparison of the *Cereibacter sphaeroide*s cellulose synthase GT domain structure with that of the *P. infestans* cellulose synthase A2 GT domain model.**

**(A)** X-ray crystallographic structure of the *C. sphaeroides* cellulose synthase BcsA-BcsB complex (PDB 5EJ1[14]). The GT domain is colored in orange, the rest of the protein is shown in transparent blue. The cellulose polymer is shown as sticks, with carbon (C) atoms colored in light grey and oxygen (O) atoms in red. **(B)** The structure of the *C. sphaeroides* cellulose synthase GT domain, represented in orange cartoon, is superposed on the model of the *P. infestans* cellulose synthase A2 GT domain, represented in light grey. Selected residues that are conserved between the two domains are shown as sticks, with C atoms colored in orange for *C. sphaeroides* and in light grey for *P. infestans*. O atoms are colored in red, and nitrogen (N) atoms in blue. Backbone and hydrogen (H) atoms are not shown.

**Figure S8. CP32 cyclic peptide docking poses on the *Pi*CESA2 GT domain.**

The model of the PiCESA2 GT domain is represented in light grey. The 11 poses of the cyclic peptide at the channel entrance are represented as sticks, with C atoms depicted in orange, O atoms in red, and N atoms in blue. The 53 poses of the cyclic peptide in the IF2–IF4 region are represented as sticks, with C atoms in yellow, O atoms in red, and N atoms in blue. The 43 poses of the cyclic peptide in the flexible region are represented as sticks, with C atoms in light blue, O atoms in red, and N atoms in blue. H atoms are not shown.

**Figure S9. Alanine scans reveal that residues R2 and W5 of CP32 are essential for its interaction with *Pi*CESA2.**

To determine which of the amino acids in CP32 are essential for its interaction with the *Pi*CESA2 protein, we introduced single alanine substitutions at each position in the peptide sequence (Ala scan) and tested them in a yeast two-hybrid experiment. **(A)** Yeast two-hybrid control assays. **(B)** Yeast two-hybrid assay with mutated CP32 variants.

**Figure S10**. **SYTOX-Green staining of *P. infestans* hyphae treated or not with CP32**.

**(A**, **B)** Hyphae of germinating sporangia stained with SYTOX Green for 30 min in the absence of CP32 peptide. **(C**, **D)** Hyphae treated as in **(A**, **B)** in the presence of CP32 (EC_50_ concentration). CP32 enhances SYTOX Green penetration into germinating sporangia, as evidenced by the increased number of stained nuclei in hyphal structures. Scale bar: 10 µm.

**Figure S11**. **Effects of CP32 on the germination of *P. infestans* sporangia**.

**(A)** Optical image showing a few sporangia, only one has successfully germinated in the presence of CP32 (EC_50_ concentration). **(B)** *P. infestans* sporangia germinated after 4 days post treatment. CP32 was removed **t**hree days after treatment. Most of the sporangia germinated, indicating that CP32 exerts a cytostatic effect by temporarily inhibiting germination. * Ungerminated sporangia. Scale bar: 15 µm.

**Figure S12. Assessment of CP32’s biological activity on microorganisms and human cells.**

**(A**–**C)** The effects of CP32 at its EC_99_ concentration were tested on liquid cultures of *Escherichia coli* (DH5α), *Agrobacterium tumefaciens* (GV3101) and *Saccharomyces cerevisiae* (AH109). Growth curves represent the average of eight biological replicates. *E. coli* was monitored for 24 h and *S. cerevisiae* and *A. tumefaciens* evaluated for over 48 h. Growth ratios were estimated based on the optical density (OD_600_). **(D)** The potential cytotoxicity of CP32 at EC_50_ and EC_99_ concentrations was evaluated using the MTT assay on HT-29 immortalized human intestinal cells. The results indicate that CP32 does not significantly affect cell viability, as evidenced by the nearly identical absorbance levels of MTT-formazan at 570 nm after 24 and 48 h of incubation. Error bars represent the standard error of the mean, based on eight biological replicates.

**Figure S13. Assessment of CP32 biological activity on tomato leaves and on hypocotyl growth in Arabidopsis.**

**(A)** Photosynthetic performance of *Solanum lycopersicum* (cv. Marmande) leaves exposed *ex vivo* to CP32 at its EC_99_ concentration for 7 days. The maximum quantum yield (Fv/Fm) and the effective quantum yield [Y(II)] of photosystem II have been assessed. Leaves treated with 1.8 g/L glyphosate served as a positive control, while water-treated leaves were used as the negative control. Representative images from 10 biological replicates are shown. CP32-treated leaves exhibit no visible damage, whereas glyphosate-treated areas display a clear reduction in photosynthetic performance. Scale bar = 1 cm. **(B)** Representative images of 3-day-old *Arabidopsis thaliana* (Col-0) seedlings grown in the dark (*n* = 35). From left to right: mock control (water), CP32 (Ec_50_ and Ec_99_), 1% (v/v) DMSO 1% (used to dissolve isoxaben), and 5 nM isoxaben. While hypocotyl growth remains unaffected by CP32 at EC_50_ and EC_99_ concentrations, it is significantly impaired in the presence of isoxaben. Scale bar: 2 mm. **(C)** Quantification of hypocotyl length from seedlings as shown in **(B)** (*n* = 35).

**Figure S14. Assessment of CP32 treatment on tomato plant growth.**

**(A**, **B)** Images of tomato plants pre-treated weekly with **(A)** water (*n* = 15) or **(B)** CP32 (100 µM; *n* = 12) for three consecutive weeks. Images were acquired after a total growth period of five weeks. No visible differences in growth were observed between control and CP32-treated plants.

**Table S1. List of peptides that specifically interact with *Pi*CESA2, identified by Y2H screening.**

All peptides were confirmed not to interact with the GAL4BD backbone. Peptides are ranked based on the strengths of their interactions with *Pi*CESA2. Antimicrobial activity was calculated based on sporangial growth inhibition (mean of six replicates ± standard deviation), compared to the untreated control. CP30 was not tested owing to difficulties in water solubilization.

| **Peptide name** | **Peptide sequence** | **Growth inhibition percentage (%)** |
| --- | --- | --- |
| CP28 | βA–VWYRGSTV | 4.17 ± 10.10 |
| CP29 | βA–MLRSYCIK | 7.53 ± 5.42 |
| CP30 | βA–RRNFYCIW | Not tested |
| CP31 | βA–DKFFESYC | 18.02 ± 5.94 |
| CP32 | βA–GRLRWLRV | 98.86 ± 0.52 |
| CP33 | βA–RRRCWLHV | 36.19 ± 9.13 |
| CP34 | βA–RLRYTCMC | 22.39 ± 5.48 |

**Table S2. Disease severity assessment for the *in vivo* assay performed on tomato plants.**

The table summarizes the results of two independent assays performed using two CP32 concentrations (50 and 100 µM).

**Table S3**. **Spectrum of action of CP32 against other phytopathogenic oomycete species (*P. capsici, P. ultimum* and *P. viticola*).**

Results are reported as mean growth inhibition (GIP) or percent protection index (PPI) compared with the untreated control ± standard deviation (See Methods). Different letters indicate significant differences between treatments, based on Kruskal-Wallis tests (*P*  ≤  0.05).

| **Treatment** | **Assayed species** | | |
| --- | --- | --- | --- |
|  | ***P. capsici*^1^**  (GIPL – %) | ***P. ultimum*^2^**  (GIPS – %) | ***P. viticola*^3^**   (PPI – %) |
| CP32 | 89.25 ± 7.35 (b) | 85.42 ± 7.28 (b) | 94.92 ± 6.45 (a) |
| Fungicide* | 100 ± 0 (a) | 100 ± 0 (a) | 100 ± 0 (a) |
| *Mandipropamid (*P. capsici* and *P. viticola*); Metalaxyl-M (*P. ultimum*)  ^1^: KW chi-squared = 8.38, df = 1, *P* value = 0.004  ^2^: KW chi-squared = 6.05, df = 1, *P* value = 0.014  ^3^: KW chi-squared = 3.03, df = 1, *P* value = 0.082 | | | |

**Table S4. PAMPA-BBB permeability classification of reference compounds used as training set.**

| **Compound** | **Pe,exp (×10⁻⁶ cm/s)** | **CNS Classification** |
| --- | --- | --- |
| Testosterone | 16.9 ± 0.8 | High |
| Diazepam | 13.0 ± 0.7 | High |
| Verapamil | 17.0 ± 0.8 | High |
| Imipramine | 11.9 ± 0.3 | High |
| Progesterone | 11.3 ± 0.5 | High |
| Oxazepam | 10.3 ± 0.8 | High |
| Chlorpromazine | 6.8 ± 0.3 | High |
| Corticosterone | 5.9 ± 0.4 | High |
| Clonidine | 5.8 ± 0.2 | High |
| Alprazolam | 5.3 ± 0.4 | High |
| Dipirydamole | 3.8 ± 0.5 | Medium |
| Loraditine | 2.5 ± 0.6 | Medium |
| Trimethoprim | 2.4 ± 0.1 | Medium |
| Chloroquine | 2.3 ± 0.1 | Medium |
| Hydrocortisone | 2.1 ± 0.3 | Medium |
| Caffeine | 1.6 ± 0.2 | Low |
| Lomefloxacin | 1.4 ± 0.1 | Low |
| Ofloxacin | 1.1 ± 0.1 | Low |
| Enoxacin | 1.04 ± 0.26 | Low |
| Atenolol | 0.78 ± 0.10 | Low |
| Dopamine | 0.24 ± 0.10 | Low |
| Norfloxacin | 0.15 ± 0.10 | Low |
| Hydrochlorothiazide | 0.35 ± 0.1 | Low |
| Theophylline | 0.12 ± 0.1 | Low |

*P*_e,exp_: Experimentally determined effective permeability values measured using the PAMPA-BBB assay.

CNS classification follows the thresholds reported by Di et al. (2003):
High permeability: *P*_e_ ≥ 4.0 × 10⁻⁶ cm/s
Medium permeability: 1.5 ≤ P_e_ < 4.0 × 10⁻⁶ cm/s
Low permeability: *P*_e_ < 1.5 × 10⁻⁶ cm/s

**Table S5. List of the peptides synthesized and used in this work.**

| **Peptide** | **Sequence** | **MW calc**  **(g/mol)** | **MW exp**  **(g/mol)** | **Purity**  **(%)** | **Amount**  **(mg)** |
| --- | --- | --- | --- | --- | --- |
| **CP28** | βA–VWYRGSTV | 1,038.09 | 1,038.6 | 90 | 3 |
| **CP29** | βA–MLRSYCIK | 1,066.3 | 1,066.0 | 90 | 4 |
| **CP30** | βA–RRNFYCIW | 1,210.4 | 1,209.9 | 90 | 7 |
| **CP31** | βA–DKFFESYC | 1,091.1 | 1,090.7 | 80 | 15 |
| **CP32** | βA–GRLRWLRV | 1,108.4 | 1,108.3 | 95 | 15 |
| **CP33** | βA–RRRCWLHV | 1,178.4 | 1,178.1 | 85 | 8 |
| **CP32-R2A:** | βA–GALRWLRV | 1,023.28 | 1,023.72 | 98 | 10 |
| **CP32-W5G** | βA–GRLRGLRV | 979.22 | 979.86 | 97 | 10 |

**Table S6. List of primer sequences used for gene cloning and gene expression analyses.**
